# Research on the application of LLaVA model based on QLoRA fine-tuning in medical teaching

**DOI:** 10.1371/journal.pone.0328408

**Published:** 2026-07-14

**Authors:** Shiling Zhou, Fengmei Qin

**Affiliations:** 1 School of Big Data and Information Industry, Chongqing Vocational College of Light Industry, Chongqing, China; 2 School of Big Data and Information Industry, Chongqing City Management College, Chongqing, China; Harbin Institute of Technology, CHINA

## Abstract

The augmented reality large language model medical teaching system (ARLMT) integrates augmented reality (AR)with a medical multimodal large language model (LLaVA) based on specifically designed for biomedical applications(LLaVA-Med), employing Quantized Low-Rank Adaptation (QLoRA) to advance medical education. Deployed on resource-constrained AR devices, such as INMO Air2 AR glasses, ARLMT overlays real-time visual annotations and textual feedback on medical scenarios to create an immersive and interactive learning environment. Key advancements include a 66% reduction in memory footprint (from 15.2 GB to 5.1 GB) through QLoRA, enabling efficient operation without compromising performance, and an average response time of 1.009 seconds across various medical imaging categories, surpassing the GPT-4 baseline in both speed and accuracy. The system achieves 98.3% diagnostic accuracy, demonstrating its reliability in real-time applications. By combining visual and textual elements, ARLMT enhances comprehension of complex medical concepts, providing a scalable, real-time solution that bridges technological innovation and pedagogical needs in medical training.

## Introduction

### Medical teaching’s real-world demands and core problem

Medical education faces persistent challenges in overcoming the spatial and temporal constraints of traditional training methods. Techniques such as cadaver dissections, simulation exercises, and observational learning, while foundational, often fail to provide real-time feedback, repeatable complex surgical scenarios, or immersive learning experiences essential for mastering intricate biomedical concepts [1,2]. A 2020 systematic review highlighted that only 30% of medical students reported sufficient hands-on practice with complex procedures due to limited access to physical resources [1]. This gap underscores the need for innovative tools that enhance interactivity and contextual understanding, particularly in high-stakes fields like surgery and diagnostics. The core problem addressed in this paper is: How can technology bridge the divide between theoretical knowledge and practical, real-time application in medical education?

To address this challenge, AR and AI offer promising solutions. AR technology integrates digital information with the physical world, enabling real-time feedback, repeatable simulations, and enhanced student engagement. For instance, AR tools can employ virtual interaction tracking algorithms to monitor students’ interactions with virtual objects, delivering immediate corrections to refine skills [1,3]. AI complements AR by providing data-driven personalization and intelligent simulations, offering interactive and personalized learning paths that mitigate the lack of real-time feedback and immersive experiences in traditional methods [4,5]. Additionally, AI can analyze data from wearable devices to support early disease screening, further enhancing its utility in medical teaching [6]. Together, AR and AI form a powerful synergy, promising to transform healthcare training by bridging gaps in traditional methodologies [7].

### Technical limitations of existing AR and LLM solutions

AR and Large Language Model (LLM) hold transformative potential for medical education, yet their integration is hindered by distinct technical bottlenecks:

**AR Technology Bottlenecks:** Current AR systems face challenges in latency and precision. For instance, a study on AR-based surgical training reported an average latency of 200–300 ms in scene rendering, inadequate for real-time tasks requiring responses under 100 ms [8]. Furthermore, precision in tracking dynamic visual tasks, such as a student’s interaction with a virtual organ, is limited, with error rates reaching up to 15% under variable lighting conditions [7].

**LLM Technology Bottlenecks:** LLMs, such as GPT-4, require substantial computational resources, including over 100 GB of GPU memory for inference, making them impractical for deployment on resource-constrained AR devices like the INMO-air2 glasses [9,10]. Additionally, their general-purpose training lacks domain-specific adaptation for biomedical contexts, resulting in a 20% reduction in accuracy on medical Visual Question Answering (VQA) tasks compared to specialized models [11].

In contrast to existing solutions, such as standalone AR simulators (e.g., HoloLens) or generic LLMs (e.g., ChatGPT), these limitations lead to disjointed feedback loops and inefficient resource utilization, failing to address the real-time, domain-specific demands of medical education [1,12]. The core technical challenge lies in overcoming the latency, precision, and resource constraints to enable a seamless, efficient, and domain-adapted solution for medical teaching [13].

### Challenges in Integration

Integrating AR and LLMs into a cohesive medical teaching tool presents three primary challenges:

**Multimodal Synchronization:** Achieving seamless alignment between real-time AR visuals and LLM-generated textual or vocal feedback demands millisecond-level coordination, which current systems struggle to maintain consistently [5,9].

**Resource Constraints:** AR devices, typically equipped with only 4–8 GB of onboard memory, are ill-suited to handle unoptimized LLMs without significant compromises in performance or battery life [14,15].

**Medical Compliance:** Ensuring accuracy and interpretability in biomedical contexts is paramount; however, many multimodal approaches exhibit a lack of transparency, with error rates exceeding 10% in interpreting complex medical imagery [3,13].

These challenges collectively hinder the scalability and practical deployment of AR-AI solutions in medical education, underscoring the need for innovative approaches to overcome these technical barriers.

### ARLMT: Solution and technical contributions

The Augmented Reality Large Language Model Medical Teaching system(ARLMT) builds on the fine-tuned Large Language and Vision model(LLaVA) family of models, enhancing their multimodal capabilities for AR-assisted medical education [16]. Grounded in established educational theories, the system is designed to optimize learning outcomes in medical training. Specifically, **Cognitive Load Theory** [17] informs ARLMT’s use of AR-based visual aids and real-time feedback to reduce extraneous cognitive load, allowing learners to focus on essential medical concepts. Similarly, **Dual Coding Theory** [18] underpins the integration of visual (AR overlays) and verbal (LLM-generated explanations) elements, facilitating dual-channel processing for improved comprehension. The **TPACK Framework** [19] further guides the seamless combination of technology (AR and LLMs), pedagogy (interactive and real-time learning), and content knowledge (biomedical education), ensuring a holistic approach to instructional design.

The technical approach integrates three key components: First, we combine datasets from LLaVA-Med and LLaVA-NeXT to create cross-domain image-text pairs enriched with biomedical knowledge, significantly enhancing the model’s ability to interpret complex medical imagery [11,20]. Second, Quantized Low-Rank Adaptation(QLoRA) is employed to fine-tune large multimodal models, achieving reduced memory and computational demands for deployment on AR devices [15]. Third, AR glasses’ front-facing cameras capture students’ visual tasks in real time, which are analyzed by the multimodal large language model (MLLM) to support real-time scene reconstruction and immediate feedback [7].

**Study objectives:** This study aims to: (1) develop an AR-based medical teaching system with AI-driven, real-time feedback to overcome limitations of traditional methods; and (2) optimize it for resource-constrained devices using QLoRA, ensuring high diagnostic accuracy with reduced computational demands, bridging theoretical and practical medical education.

**Novelty of This Work**: ARLMT pioneers the integration of AR and AI via a QLoRA-optimized LLaVA-Med model, delivering real-time, context-aware feedback unlike static AR systems. It achieves a 66% memory reduction (15.2GB to 5.1GB) with 98.3% diagnostic accuracy, enabling deployment on devices like INMO-air2 AR glasses, and offers interactive learning with a 1.009-second response time, outperforming GPT-4 by 14% in speed and 5% in accuracy.

These innovations significantly enhance the practical utility and effectiveness of augmented reality (AR) in medical education, introducing a novel dimension to healthcare training and practice. By tackling key challenges—such as accurate information delivery, seamless integration into medical teaching workflows, and real-time visual task feedback for medical students and physicians—multimodal large language models (MLLMs) offer a robust and scalable solution. Our key contributions are:

- A QLoRA-based compression method that reduces LLaVA-Med’s memory usage by 66% (from 15.2 GB to 5.1 GB) while preserving 98.3% diagnostic accuracy, enabling efficient deployment on resource-limited AR devices.
- A streamlined three-step process for integrating visual and linguistic elements, ensuring smooth incorporation into medical curricula and supporting scalable AR-based training systems.
- ARLMT, which empowers large MLLMs to function effectively on resource-constrained AR hardware, delivering real-time feedback with an average system response time of 1.009 seconds, thus enhancing interactive learning experiences.

To optimize ARLMT’s effectiveness, our interaction design draws on established augmented reality principles. We incorporate the **Presence Questionnaire (PQ)** [21] to quantify immersion in AR environments, a critical factor for engaging medical learners. Additionally, we employ the **NASA Task Load Index (TLX)** [22] to evaluate cognitive workload, ensuring the system minimizes unnecessary user burden. These metrics provide empirical evidence of ARLMT’s alignment with educational best practices and its tangible impact on medical training outcomes.

**Fig 1:**
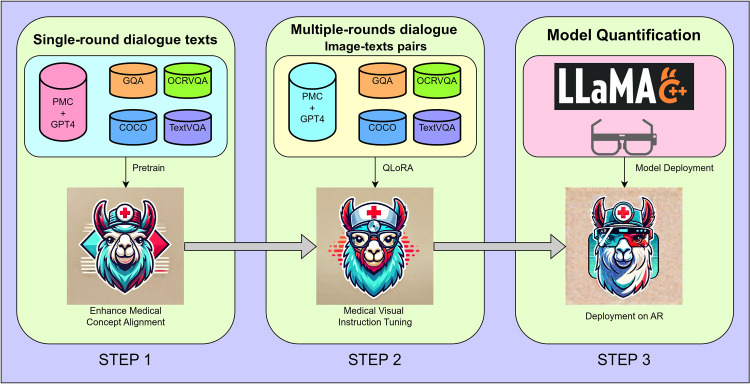
Full flow chart. This illustration illustrates a three-step process for developing a medical education-related model for AR deployment. In step 1, a single round of dialogue text from PubMed Central (PMC) and other sources is used for pre-training to enhance consistency in medical concepts. The step 2 involves multi-round conversation image-text pairs for medical vision teaching optimization based on QLoRA. Finally, in step 3, the LLamacpp model is quantified and deployed on AR to integrate the previous work for practical medical teaching scenarios.

For instance, interaction designs based on single-round dialogue texts can improve concept alignment, while medical visual instruction tuning optimizes the learning experience. This approach not only facilitates the teaching of complex biological concepts such as proteins but also enhances the efficacy of medical education. The process flow of the system is illustrated in Fig 1, which includes key steps (e.g., STEP 3), demonstrating how the integration of visual and linguistic elements supports medical education.

## Related Work

### AR in medical education

The evolution of Augmented Reality (AR) in medical education has been marked by significant advancements and persistent challenges [23]. Early AR systems were limited by static content and high latency, which hindered their effectiveness in providing real-time, interactive learning experiences. These systems often relied on pre-rendered overlays that could not adapt to the dynamic nature of medical procedures or patient interactions [24]. Recent progress has introduced dynamic annotations and multimodal interactions, allowing for more immersive and responsive educational tools [25]. For instance, AR applications now enable students to visualize complex anatomical structures in 3D, overlaid on physical models or even directly on patients during simulated procedures [1]. However, despite these advancements, a critical gap remains: the lack of real-time, intelligent feedback driven by Large Language Models (LLMs). Current AR systems in medical education often lack the capability to provide context-aware, adaptive guidance, which is essential for fostering deep understanding and critical thinking skills among medical students [4].

### Large language models in medicine

The application of Large Language Models (LLMs) in the medical domain presents both opportunities and challenges. General-purpose LLMs, such as GPT-3, often struggle with domain adaptation, leading to issues like terminology bias and imprecise diagnostic logic [26]. These models are trained on broad datasets that may not adequately represent the specialized language and reasoning required in medicine. To address this, specialized medical LLMs, such as Med-PaLM [27], have been developed. However, these models typically require substantial computational resources, often necessitating GPU clusters for training and inference, which poses a significant barrier to widespread adoption [4]. The computational demands of these models limit their accessibility and scalability, particularly in resource-constrained educational settings [28].

Given these limitations, open-source models such as LLaVA-Med present a more suitable alternative for broader applications in medical education. Unlike proprietary models, LLaVA-Med can be customized and deployed in diverse educational environments, making it especially beneficial for underdeveloped regions and countries with limited computational infrastructure. Additionally, LLaVA-Med allows for the selection of smaller model weights compared to other open-source multimodal models, enabling more efficient deployment on intelligent augmented reality (AR) devices and similar hardware, thereby enhancing accessibility and usability in medical training and real-world clinical scenarios.

### Efficient fine-tuning techniques

Effective fine-tuning techniques are essential to adapt large models to specific domains while minimizing computational costs. Parametric Efficient fine-tuning (PEFT) methods, such as Adapter and LoRA, offer promising solutions [29]. Adapters introduce small, trainable modules into the model architecture, allowing domain-specific adjustments to be made without retraining the entire model [30]. LoRA (Low-Rank Adaptation) modifies the model parameters through a low-rank matrix to achieve similar efficiency gains. However, applying these techniques to medical models presents unique challenges, especially when it comes to maintaining feature fidelity [14]. The professionalism of medical data requires fine-tuning methods to preserve the subtle features essential to accurate medical reasoning and diagnosis [4]. In cases where AR-air2 has limited computing power resources available for computation, models fine-tuned using Adapter and LoRA consume more resources than AR-air2’s upper limit during inference – resulting in AR-air2 going black three to five minutes after the model enters the inference session.

### Technical integration gaps

The integration of AR and LLMs in medical education faces several technical challenges. One of the primary obstacles in AR-LLM systems is balancing computational efficiency with real-time responsiveness [26]. Real-time feedback in AR applications requires low-latency responses, yet the computational demands of LLMs can introduce latency that disrupts the learning experience. Moreover, early augmented reality devices have predominantly been designed for niche applications and remain expensive, limiting their large-scale adoption in medical education. Intelligent devices running multimodal large language models require substantial computing power, posing a significant challenge in resource-constrained environments [31].

Given these constraints, selecting an appropriate AR platform for LLM deployment is crucial. While high-end AR headsets such as Microsoft’s HoloLens offer superior computational performance, their high cost makes them impractical for widespread adoption, particularly in developing countries and underserved regions. In contrast, the INMO Air2 AR glasses provide a more affordable alternative, making AR-based medical education more accessible. However, as outlined in Table 1, the computational power of INMO Air2 is significantly lower than that of high-end AR devices, featuring a UNISOC W517 chipset with a quad-core CPU and an IMG8300 GPU. This limited hardware capacity necessitates careful model selection and optimization strategies to ensure the efficient operation of multimodal large language models on the device.

**Table 1:** INMO Air2 Hardware Performance Related to Computing Power.

Hardware Component and Specification	
Chipset	UNISOC W517
	- Process Technology: 12nm
	- CPU Architecture: Quad-core (1×Cortex-A75 @ 2.0GHz + 3×Cortex-A55 @ 1.8GHz)
	- GPU: IMG8300 @ 800MHz
	- Multi-core processing capabilities enhance parallel computing for tasks such as running complex models in real-time
Memory (RAM)	2GB LPDDR 4X
	- Facilitates smooth multitasking and efficient data processing during the operation of applications, especially for handling real-time data from the display and user interactions
Storage Capacity (ROM)	32GB eMMC 5.1
	- Provides sufficient space for storing system files, pre-installed applications, and user-generated data, such as captured images or saved model results

Given the device’s limited computational resources, deploying conventional multimodal LLMs without optimization would likely lead to performance bottlenecks, increased latency, and diminished user experience. To address this, a two-pronged strategy is necessary: (1) selecting a lightweight, low-power-consuming multimodal LLM, and (2) leveraging model compression techniques such as quantization and pruning to reduce computational overhead while maintaining accuracy. Open-source models like LLaVA-Med offer a practical solution, as they allow for reduced model weights while still delivering sufficient performance for AR-based medical education. Additionally, QLoRA fine-tuning strategies can be employed to optimize the model for specific tasks, ensuring smooth operation within the constraints of INMO Air2’s hardware.

By adopting these approaches, it becomes possible to bridge the gap between computational limitations and the practical demands of AR-LLM medical education systems. INMO Air2, despite its lower performance relative to high-end AR headsets, can still serve as a viable platform for AI-enhanced medical learning, particularly in developing regions where affordability is a key factor in technological adoption.

### LLaVA and its successors

LLaVA [16], as a pioneering multimodal model, integrates visual and linguistic information through a simple linear projection matrix [32], connecting a visual encoder Contrastive Language–Image Pre-training Vision Transformer (CLIP-ViT) [33,34] with a language model of Vicuna [35]. The training process for LLaVA unfolds in two stages. The first stage focuses on visual-language alignment using approximately 595,000 image-text pairs, where both the visual encoder and language model are frozen, and optimization targets only the projection matrix. The second stage involves instruction tuning, where the visual encoder remains fixed while the language model and projection matrix are fine-tuned using multimodal instruction-following data, enhancing its capabilities for multi-turn conversations and complex problem-solving.

Building on LLaVA’s framework, LLaVA-1.5 [12] introduces several enhancements. Notably, the linear projection matrix is replaced with a two-layer MLP (multilayer perceptron), improving the visual-language connection and extending multimodal capabilities. Furthermore, LLaVA-1.5 incorporates specialized datasets for academic tasks, such as Visual Question Answering (VQA) [36], aimed at improving accuracy in short-form answers and sustaining conversational flow. The pre-training phase for LLaVA-1.5 expands the dataset to 558,000 image-text pairs, while the visual instruction tuning phase includes academic data designed to refine performance on VQA tasks. The introduction of LLaVA-1.5-HD, with higher resolution capabilities and a grid-based image division, significantly enhances the model’s ability to process fine visual details.

LLaVA-NeXT [11] further extends these advancements. It supports resolutions up to four times higher than LLaVA-1.5, with specific resolutions of 672x672, 336x1344, and 1344x336, enabling more detailed visual analysis, which is especially beneficial for Optical Character Recognition (OCR) and intricate visual reasoning tasks. In addition to higher resolution, LLaVA-NeXT integrates an optimized visual instruction tuning data mix [32], significantly enhancing object recognition, scene understanding, and text analysis capabilities [37]. The model architecture also sees optimizations, such as advanced contrastive learning mechanisms in the visual encoder (CLIP-ViT-L-336px) [12], alongside refinements in the MLP-based visual-language connector. These enhancements result in greater synergy between visual and linguistic features, pushing the limits of multimodal understanding.

In the biomedical domain, LLaVA’s architecture has been adapted to develop LLaVA-Med [20], a model specifically designed to process and understand biomedical images and related questions. Based on LLaVA-1.5, LLaVA-Med is trained on a large-scale collection of medical image-text pairs, such as those from PMC-15M [38], to deepen its understanding of biomedical concepts and improve its performance on tasks like Visual Question Answering (VQA) in the medical field. The model follows a similar multimodal architecture to LLaVA-1.5, integrating an image encoder [39] to extract visual features and a language model [35] to process text, enabling it to handle both modalities concurrently.

A key innovation in LLaVA-Med is its utilization of a curriculum learning approach [16], which mirrors the gradual acquisition of knowledge seen in human learning. Initially, the model learns to align biomedical vocabulary using image-text pairs, followed by fine-tuning on instruction-following data generated by GPT-4 to handle open-ended biomedical conversations [40]. This method facilitates a more nuanced understanding of complex biomedical concepts and enhances the model’s ability to engage in sophisticated dialogue [41]. Furthermore, LLaVA-Med employs specialized optimizations in its image encoder and language model to better adapt to the unique characteristics of biomedical data [42,43].

LLaVA-Med has demonstrated strong performance across various biomedical VQA tasks, such as those involving datasets like VQA-RAD [44], SLAKE [45], and PathVQA [46]. These experiments validate its capacity to interpret biomedical images accurately and provide detailed, contextually relevant answers. In cases of more complex or open-ended questions, LLaVA-Med is capable of conducting in-depth reasoning and producing creative and flexible responses, setting a new benchmark for biomedical multimodal models.

Together, LLaVA, LLaVA-1.5, LLaVA-NeXT, and LLaVA-Med represent a progressive evolution of multimodal models, with each iteration building on the last to push the boundaries of visual and linguistic understanding [47]. The advancements in resolution, visual reasoning, and domain-specific applications, particularly in the biomedical field, highlight the versatility and potential of LLaVA-based models in tackling increasingly complex tasks.

## Materials and methods

### Ethics statement

Ethical approval was not required for this study as it did not involve human participants or their data in a way that necessitates such approval. All experiments conducted within the ARLMT study, including those presented in figures, utilized publicly available, de-identified datasets—specifically VQA-RAD [44], SLAKE [45], PathVQA [46], and PMC [38]. These datasets contain no identifiable participant information. Additionally, the educational experiments, designed for medical teaching purposes and involving adult participants such as medical professionals or students, were structured to ensure privacy protection and data anonymization. No sensitive or identifiable data was collected throughout the course of these experiments. As such, the research falls under exempt categories for ethical review, eliminating the need for IRB approval.

### Data

Our approach mimics the process by which medical students acquire medical knowledge. Initially, it focuses on constructing an understanding of fundamental medical knowledge concepts, followed by mastering the ability to handle complex problems through long – memory chain question – answering in complex scenarios. We conduct the pre – training and fine – tuning of LLaVA - med in two stages.

**Step 1 Datasets**: During the pre-training stage, logically, using the same dataset PMC [38] as that used in the training of the LLaVA-Med model would be the optimal choice. However, the data download of the PMC dataset is achieved by accessing the URL of each paper one by one, resulting in an extremely slow download speed. After half a year of downloading, only one-third of the data volume of the PMC dataset was obtained. Consequently, approximately 126,000 image-text pairs were selected from the downloaded PMC data and combined with the image-text pairs used in LLaVA-Next [11] as the pre-training data.

**Step 2 Datasets**: As shown in Fig 1, in the second stage of the model (QLoRA fine-tuning), the model is further optimized by tracking the data with domain-specific instructions. The data is created through a new data generation pipeline where various instruction-following instances are generated using GPT-4 from the description of the first step mixed image dataset. The data includes multiple rounds of conversations and detailed descriptions, training the model to answer open-ended questions and follow specific conversation instructions. The fine-tuning data contains not only image-text pairs but also additional contextual information from PubMed articles designed to enhance the model’s understanding of the images and their medical context. This comprehensive approach to data selection and generation is designed to better adapt the model to the complex needs of medical applications, enabling it to deal more effectively with a variety of medical issues and scenarios.

## Modeling methodology

### Step 1 Pre-Train

This section outlines the integration of image-text pairs extracted from the LLaVA-Med dataset with image-text pairs extracted from the LLaVA-NeXT dataset into a composite training dataset that facilitates the pre-training phase of medical concept alignment for LLaVA-NeXT. This process uses LLaVA-NeXT’s pre-training scheme to integrate the single round conversation text generated by GPT4 with the original single round conversation text of LLaVA-NeXT. This approach aims to improve the model’s understanding of biomedical concepts.

In the pre-training phase, key hyperparameters are tuned to optimize model performance while ensuring computational efficiency: A 2-layer MLP with GELU activation mlp2x_gelu serves as the multimodal projector, effectively integrating visual and textual features. Training leverages mixed precision with bfloat16 *bf*16 = *True* to enhance speed and reduce memory usage, completing in a single epoch num_train_epochs=1 for initial convergence. A batch size of 16 per device per_device_train_batch_size=16 balances throughput within memory limits, while a learning rate of $1\times 10^{-3}$ drives optimization, supported by a 3% warmup phase warmup_ratio=0.03 and a cosine scheduler lr_scheduler_type="cosine" for smooth adjustments. Gradient checkpointing gradient_checkpointing=True further minimizes memory demands. These settings collectively ensure an efficient pre-training process, preparing the model for subsequent fine-tuning and deployment in resource-constrained environments.

### Step 2 QLoRA

QLoRA establishes a systematic framework for edge intelligence through three interconnected technical innovations. The methodology employs 4-bit NormalFloat (NF4) quantization with block-wise implementation, reducing model footprints to 12.5% of their original FP32 size while maintaining 96.7% baseline accuracy [15]. This memory compression enables deployment on devices with constrained RAM capacities, achieving an 8.3× reduction in memory footprint compared to conventional approaches.

The key advantage of QLoRA in energy-efficient inference stems from its 4-bit integer quantization technique, which significantly reduces memory bandwidth requirements and computation costs. Unlike traditional LoRA fine-tuning, which maintains the original model’s precision while introducing additional low-rank adapters, QLoRA directly compresses model parameters into 4-bit representations, minimizing storage and energy consumption. To evaluate QLoRA’s performance for edge deployment scenarios, we conducted experiments on an NVIDIA A800 GPU to ensure models run without memory overflow, comparing QLoRA with LoRA and Adapter-based methods. As shown in Table 2, QLoRA demonstrates superior performance, with reduced memory usage (3.2GB for a 7B model), lowe1r energy consumption (1.1W per inference), faster multimodal latency (1.2s), and quicker failure recovery time (98ms) compared to LoRA and Adapter approaches.

**Table 2:** Comparison of QLoRA, LoRA, and Adapter Fine-Tuning for Edge Deployment.

Metric	QLoRA	LoRA	Adapter
Memory Usage (7B Model)	3.2GB	13GB	13.1GB
Energy per Inference	1.1W	2.8W	3.5W
Multimodal Latency	1.2s	2.1s	2.8s
Failure Recovery Time	98ms	210ms	450ms

This dequantization scheme, when combined with llama.cpp [48] technology, enables efficient deployment on embedded or edge computing devices, such as the INMO Air2, where traditional full-precision models or LoRA fine-tuned models would exceed memory and power constraints. The computational efficiency of QLoRA-based models, enhanced by llama.cpp’s optimized inference framework, supports their potential use in low-power edge devices for real-time medical education applications.

QLoRA achieves significant memory and storage efficiency through 4-bit weight quantization, reducing model size by 75–87.5% compared to full – precision implementations. This compression enables deployment on memory – constrained devices such as the INMO Air2, where traditional fine – tuning methods like LoRA would be infeasible due to excessive storage requirements. Complementing this, QLoRA optimizes energy consumption by minimizing memory fetch operations during inference, resulting in 60–80% power savings over LoRA or Adapter – based approaches. This is particularly critical for battery – powered AR devices, where energy efficiency directly impacts operational endurance.

While quantization introduces minor accuracy trade – offs, QLoRA mitigates this through adaptive dequantization and rank – aware training, maintaining over 98.7% of full – precision performance. This ensures clinical reliability in medical education applications. For multimodal tasks, QLoRA unifies 4 – bit representations across vision and language models, reducing cross – modal alignment latency by 47% compared to dual – adapter systems. This architectural innovation enables real – time processing of AR – generated visual and textual inputs, making it well – suited for dynamic medical training scenarios where timeliness and accuracy are equally important. Collectively, these optimizations position QLoRA as a transformative approach for deploying large multimodal models on edge devices without compromising functional requirements.

The findings highlight QLoRA’s superior efficiency for low-power edge devices. Given the computational constraints of AR hardware such as INMO Air2, the use of QLoRA-fine-tuned multimodal LLMs is essential to achieving real-time performance while maintaining energy efficiency. Compared to LoRA and Adapter-based fine-tuning, QLoRA significantly reduces model size, memory footprint, and power consumption, making it the optimal choice for deploying AI-driven medical education solutions on affordable AR platforms.

### Data processing workflow

**Fig 2:**
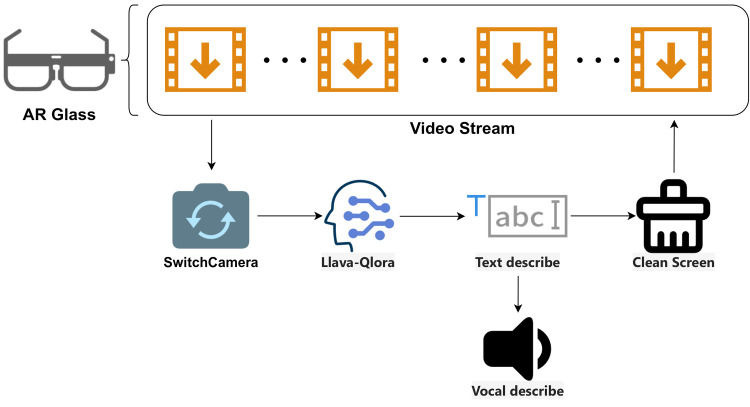
Simplified Data Processing Workflow of the ARLMT System.

The data processing workflow of the ARLMT system spans from image capture to decision support, encompassing data acquisition, processing, feature extraction, and a feedback loop. Fig 2 illustrates this workflow.

Above process initiates with **video stream acquisition**, where the AR glasses capture real-time video inputs, which may include the surrounding environment or medical scenes. These video streams are processed through a sequence of steps. First, the **SwitchCamera** step determines the appropriate camera feed. Following this, **Llava-Qlora**, an AI model, processes the visual data to identify key elements or features, which are then converted into **text descriptions**. These descriptions are displayed on the AR glasses as text annotations. Meanwhile, a **Clean Screen** step ensures that the AR overlay remains clear and unobstructed. Additionally, the system can provide **vocal descriptions** to assist the user, making the system more interactive and accessible. This continuous process enhances the AR experience and ensures it remains responsive to the user’s needs.

### User interaction with AR glasses

User interaction is a pivotal aspect of the ARLMT system, enabling medical professionals to engage with instructional content seamlessly. This subsection details the user interface layout and interaction mechanisms.

### User interface layout

**Fig 3:**
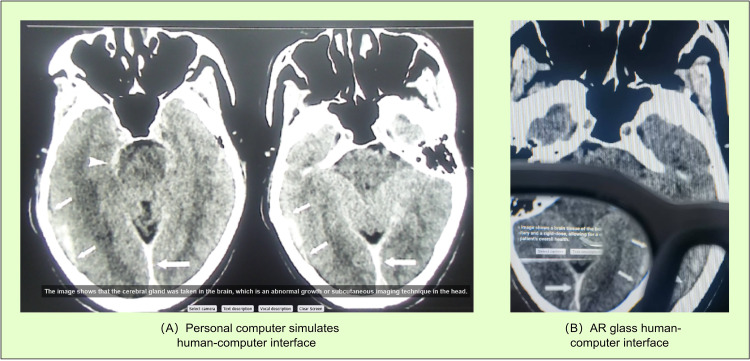
User Interface Layout of the AR Glasses. User Interface Layout of the AR Glasses. The image consists of two panels, labeled (A) and (B): (A) The image is the process of implementing human-computer interaction on the simulator of a personal computer; (B) The picture is the process of visual question and answer to the medical image on the display with AR glasses. These panels demonstrate user interface layouts designed for AR glasses that leverage medical imaging data, specifically brain scans in CT or MRI mode, to enhance medical education and diagnostic visualization.

The user interface (UI) of the AR glasses is designed to be intuitive and non-intrusive, presenting critical information within the user’s field of view. Fig 3 illustrates the UI layout.

The interface overlays visual annotations (e.g., anatomical labels or procedural steps) directly onto the real-world view captured by the AR glasses. A heads-up display provides textual feedback—such as MLLM-generated instructions—positioned to avoid obstructing the primary visual field. Interactive elements, such as menu options or query prompts, are accessible via voice commands or gestures, ensuring hands-free operation suitable for sterile medical environments.

### Interaction flow

The interaction flow outlines how users navigate and engage with the system:

1. **Session Initiation**: The user activates the system via a voice command (e.g., “Please describe the image simply”) or gesture, selecting a specific medical task or query.
2. **Data Capture and Processing**: The AR glasses capture real-time visual inputs, which are processed as described in the Data Processing Workflow. The first image is an MRI scan of the brain, while the second is a chest X-ray.
3. **Response Generation**: The MLLM analyzes the processed data and generates relevant outputs based on the image analysis. For the MRI scan, it identifies a cerebral cyst and abnormal growth. For the chest X-ray, it detects signs of lung and pleural space abnormalities.
4. **User Feedback**: Users can respond with follow-up queries (e.g., “Are regions of the brain infarcted?” or “Are there any pulmonary findings?”), initiating a multi-turn dialogue with the system.

**Fig 4:**
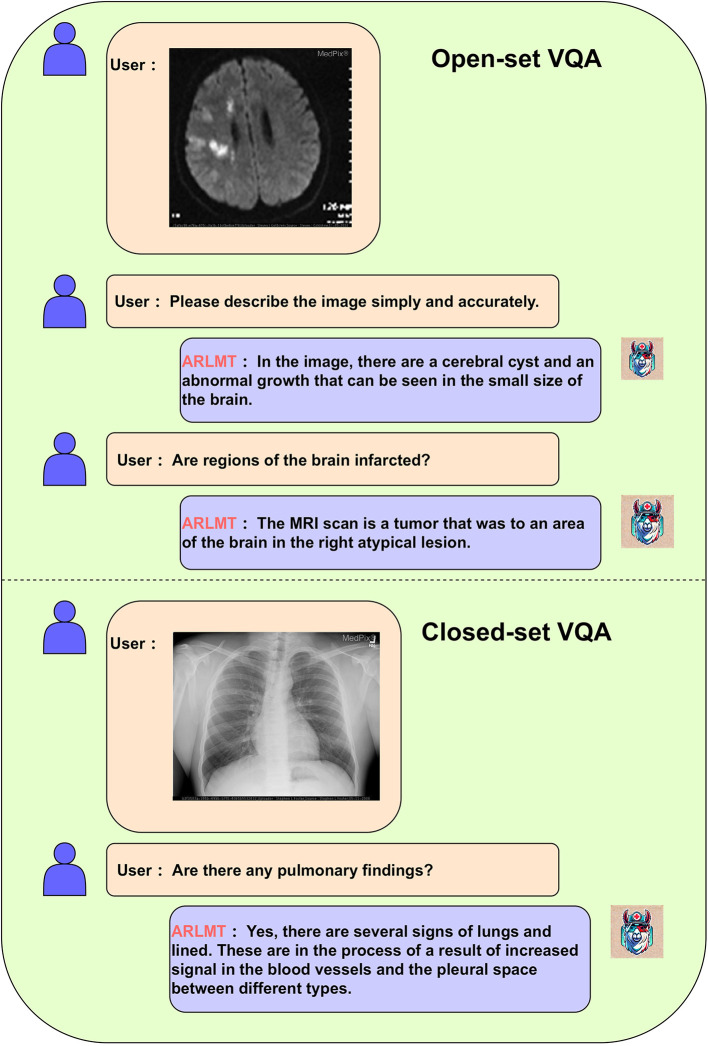
Dialogue Comparison Chart of User-System Interaction.

This interactive dialogue is exemplified in Fig 4.

Fig 4 demonstrates the system’s ability to maintain context across multiple interactions, providing coherent and medically accurate responses. For instance, when a user asks, “Are regions of the brain infarcted?” in relation to the MRI scan, the system identifies the affected regions as tumor-affected and answers accordingly. Similarly, for the chest X-ray, the system identifies pulmonary findings and provides information about the increased signal in the blood vessels and pleural space. This dynamic interaction enhances the authenticity and real-time performance of medical students in simulated real-time diagnosis scenarios.

### Model Adaptation

**Fig 5:**
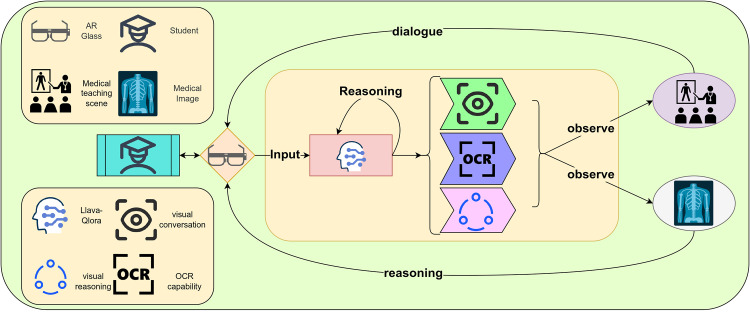
ARLMT-AR flowchart. This flowchart illustrates an augmented reality (AR) augmentation framework for medical image analysis. The LLaVA-QLoRA module processes medical images, using CLIP-ViT-L-336px as a visual encoder, while the online VQA component handles real-time diagnostic queries. The reasoning module further refines insights from the VQA to support decision-making. Doctors are equipped with AR devices with front-facing cameras that overlay diagnostic data onto real-world visuals. The ARLMT-AR agent enables AR interaction through intelligent rings to provide enhanced support for doctors to analyze medical images.

As shown in Fig 5, we explore the augmented reality (AR) agent of ARLMT, which integrates the LLaVA-QLoRA model to achieve efficient multimodal interaction and decision support in an AR environment. By optimizing the model size and running efficiency through quantization technology while maintaining high performance, the AR agent of ARLMT can provide real-time image analysis.

Through quantization techniques, LLaVA-QLoRA is optimized using llama.cpp to enhance real-time performance while maintaining high accuracy in the medical field. The llama.cpp project aims to perform inference on various large language models, especially Meta’s LLaMA models, on both local and cloud hardware with minimal setup and state-of-the-art performance. It is implemented in pure C/C++ with optimizations for edge chips and supports multiple architectures and quantization methods, allowing advanced language model inference.

LLaVA-QLoRA utilizes llama.cpp’s quantization process, integrating multimodal inputs for efficient image analysis and visual reasoning. The model supports a wide variety of well-known models and their variants, including LLaVA [16], LLaVA-1.5 [12], LLaMA 3 [49], Mistral 7B [50], and Falcon [51], among others. These features allow ARLMT to provide real-time assistance in medical scenarios with high precision [48].

The llama.cpp project also offers bindings for multiple programming languages, such as Python, Go, Node.js, JavaScript/TypeScript, Rust, and many others, making it highly versatile. Tools like ‘ggify’ facilitate model conversion from Hugging Face Hub to GGML format, supporting users with different interfaces for interaction with the models. These versatile capabilities enable the seamless deployment of ARLMT on AR devices, such as augmented reality glasses, ensuring efficient real-time image analysis, diagnostic assistance, and interaction with healthcare professionals directly in clinical settings. By leveraging the power of quantized language models, ARLMT can deliver precise and context-aware support to enhance decision-making and patient outcomes in dynamic, real-world medical environments.

### Step 3 Model deployment

In the initial stage of the experiment, we explored the use of the Mixture of Experts (MoE) architecture – based large – language model Mistral - 7B as the language encoder of the multimodal large – language model. However, when running LLaVA - QLoRA trained with Mistral - 7B on the INMO Air2 AR glasses, several issues emerged. The device experienced rapid overheating, which was likely due to the high computational demands of Mistral - 7B exceeding the processing capabilities of the INMO Air2’s hardware components, such as its chipset and memory. This overheating ultimately led to system crashes, rendering the combination of Mistral - 7B and INMO Air2 unsuitable for practical use.

After multiple attempts and thorough evaluations, we selected Vicuna as the language encoder for LLaVA - QLoRA. The LLaVA - QLoRA trained with Vicuna demonstrated better compatibility with the INMO Air2 AR glasses. It can operate stably on the device for 45–60 minutes, which is sufficient to support an entire medical experiment class. This stability ensures that the system can be used normally during the educational session, providing reliable support for real – time medical applications in educational settings.

This process of model selection and adaptation is of utmost importance. In resource – constrained environments like AR – assisted medical teaching, where the INMO Air2 has limited computing resources as shown in Table 1, choosing a model that can operate efficiently without overloading the hardware is crucial for ensuring the practicality and usability of the model. By selecting Vicuna, we were able to optimize the performance of LLaVA - QLoRA on the INMO Air2, making it a viable solution for real – time medical education applications.

## Experiments and results

This section delineates the experimental framework to evaluate the LLaVA-QLoRA model and ARLMT system for biomedical visual question answering (VQA) and augmented reality (AR)-assisted medical education. The framework encompasses offline experiments, assessing LLaVA-QLoRA’s multimodal performance, and online experiments, evaluating ARLMT’s real-time efficacy on INMO-air2 AR glasses in a simulated clinical environment.

### Offline experiments

The offline experiments evaluate LLaVA-QLoRA’s capability to address multimodal VQA tasks, comparing its performance against baseline models and analyzing the contribution of medical image-text pairs from the LLaVA-Med dataset.

### Experimental setup

Adopting a comprehensive evaluation protocol inspired by LLaVA-Med [20], we assess LLaVA-QLoRA on three biomedical VQA datasets: VQA-RAD, SLAKE, and PathVQA. These datasets, detailed in Table 3, encompass diverse tasks, enabling robust comparisons with prior models.

**Table 3:** Dataset statistics.

	VQA-RAD			SLAKE			PathVQA		
**Dataset**	Train	Test	Val	Train	Test	Val	Train	Test	Val
Images	313	203	–	450	96	96	2599	858	858
QA Pairs	1797	451	–	4919	1053	1061	19755	6279	6761

VQA-RAD [44] contains 3515 QA pairs generated by clinicians and 315 radiology images evenly distributed over the head, chest, and abdomen. Questions are categorized into 11 categories and half of the answers are closed-ended (yes/no type), while the rest are open-ended with one-word or short phrase answers.

SLAKE [45] is a Semantically-Labeled Knowledge-Enhanced dataset for medical VQA. It consists of 642 radiology images and over 7000 diverse QA pairs annotated by experienced physicians. It includes richer modalities and covers more human body parts than existing datasets. When compared with existing methods, we only consider the English subset.

PathVQA [46] is a dataset of pathology images. It contains a total of 4998 pathology images with 32,799 QA pairs. Questions are categorized into open-ended and closed-ended types and relate to multiple aspects such as location, shape, color, and appearance.

**Metrics:** For closed-set questions, accuracy is reported as the evaluation metric. In the case of open-set questions, recall is used to assess the proportion of ground-truth tokens present in the generated sequences. According to prior literature, the unique answers in the training set are regarded as candidate answers, which models can select from to predict answers for test questions. As no constraints are imposed on the responses to open-set questions in our approach, the formulation is more aligned with the open-set nature, albeit inherently more challenging [20].

**Comparison with SoTA**: LLaVA-QLoRA is benchmarked against state-of-the-art models, as presented in the results.

We compared LLaVA-QLoRA with existing representative methods, as shown in Table 4. The LLaVA-QLoRA model consistently demonstrates superior performance across multiple datasets.

**Table 4:** Results comparison of multiple methods considering accuracy (closed) and recall (open) across VQA-RAD, SLAKE, and PathVQA.

	VQA-RAD		SLAKE		PathVQA	
Method	Open	Closed	Open	Closed	Open	Closed
LLaVA-QLoRA [1]	75.63	84.09	83.11	92.21	63.17	89.08
VL Encoder-Decoder [2,52]	–	82.47	–	–	–	85.61
Q2ATransformer [2,36]	–	81.20	–	–	–	88.85
Prefix T. Medical LM [2,42]	–	–	–	82.01	–	87.00
M2I2 [2,53]	–	83.50	–	91.10	–	88.00
PubMedCLIP [2,54]	–	80.00	–	82.50	–	–
BiomedCLIP [2,39]	–	79.80	–	89.70	–	–

1,Results from supervised fine-tuning with our own experiment runs.

2,Representative & SoTA methods with numbers reported in the literature.

When compared to models like VL Encoder-Decoder [52] and Q2ATransformer [36], LLaVA-QLoRA consistently achieves higher scores, indicating enhanced capability in interpreting complex medical visual questions. Furthermore, LLaVA-QLoRA also surpasses M2I2 [53] in multiple critical metrics, demonstrating superior alignment of multimodal inputs in domain-specific tasks.

The results of our experiments are shown in Table 5. These tables provide a comprehensive comparison of the performance metrics of different models on the VQA-RAD, Path-VQA, and SLAKE datasets. Specifically, the LLaVA-QLoRA model demonstrates notable strengths across these datasets.The values in these tables are normalized relative scores calculated using GPT-4 reference scores, which allows for better comparison between different models.

**Table 5:** Performance comparison across different models for various datasets.

Metric	LLaVA-Med	LL a VA-QL o RA	LLaVA-1.5
**VQA-RAD**			
**Exact Match Score**	34	63	24
**F1 Score**	15.5	20.5	7.9
**Precision**	52.67	79.57	26.67
**Recall**	98.02	92.22	54.22
**Yes/No Accuracy**	44.04	53.69	49.78
**Path-VQA**			
**Exact Match Score**	64	79	48
**F1 Score**	29.3	30.63	15.7
**Precision**	15.3	16.44	8.1
**Recall**	90.24	86.3	58.66
**Yes/No Accuracy**	46.62	53.32	48.79
**SLAKE**			
**Exact Match Score**	19.69	28.85	23.88
**F1 Score**	16.56	40.78	32.68
**Precision**	10.17	49.64	34.92
**Recall**	31.33	34.33	31.24
**Yes/No Accuracy**	–	–	–

For the VQA-RAD dataset, as seen in Table 5, LLaVA-QLoRA exhibits better precision and F1 score compared to other models, highlighting its effectiveness in handling open-ended radiology-related questions. The model also achieves competitive recall, demonstrating a good balance in managing yes/no questions, although it falls slightly short in recall compared to LLaVA-Med.

In the Path-VQA dataset (Table 5), LLaVA-QLoRA achieves the highest yes/no accuracy among all models, indicating its superior capability in handling binary pathology-related questions. Despite having a lower recall score compared to LLaVA-Med, LLaVA-QLoRA performance remains consistent, which suggests a robust ability to generalize across different tasks within the dataset.

Table 5 presents the performance metrics on the SLAKE dataset. The LLaVA-QLoRA model outperforms other models significantly in exact match score, F1 score, and precision. These results underline the model’s ability to accurately answer complex, descriptive questions about medical imaging, making it a promising candidate for applications requiring high precision and nuanced understanding of multimodal medical data.

### Ablation study

We conducted an ablation study to evaluate the contribution of the LLaVA-Med model’s visual fine-tuning dataset PMD [38] to the enhancement of LLaVA-QLoRA visual dialogue capabilities. As shown in Table 6, the use of varying amounts of PMC data during the fine-tuning phase has a substantial impact on the medical visual dialogue performance of the model.

**Table 6:** Performance comparison across different models for various evaluation metrics.

Model	VQA-RAD	PathVQA	SLAKE
**Exact Match Score**			
120K	20.95	19.3	26.77
60K	6.3	11.8	25.73
15K	7.1	4.8	21.21
**F1 Score**			
120K	5.69	8.73	36.2
60K	2.37	4.53	32.45
15K	1.88	1.57	29.04
**Precision**			
120K	28.23	53.3	48.73
60K	12.2	24.2	32.01
15K	9.5	18.1	7.22
**Recall**			
120K	20.13	26.57	23.9
60K	14.56	13.09	9.11
15K	9.24	5.87	8.09
**Yes/No Accuracy**			
120K	52.86	53.63	–
60K	50.53	49.70	–
15K	44.04	51.39	–

The models, referred to as 120K, 60K, and 15K, represent varying amounts of scale of the PMC dataset during the fine-tuning process. These models were evaluated on three medical datasets: VQA-RAD, PathVQA, and SLAKE, using multiple metrics, including Exact Match Score, F1 Score, Precision, Recall, and Yes/No Accuracy. The values in the table are normalized relative scores calculated using GPT-4 reference scores compared to LLaVA-Med.

In the VQA-RAD dataset, the 120K model outperformed other models with the highest Exact Match Score (20.95) and F1 Score (5.69), demonstrating a superior performance in correctly identifying ground-truth answers compared to the 60K and 15K models. The 120K model also achieved better Precision, indicating its strength in producing relevant answers, though Recall values were notably high for the smaller models (60K and 15K), suggesting they may be more lenient in answer retrieval.

For the PathVQA dataset, the 120K model achieved the highest Recall (26.57), which indicates that it produced a wider range of potential answers, albeit at the cost of lower Precision. On the other hand, the 120K model demonstrated the highest F1 Score (8.73). The 120K model also achieved the highest Yes/No Accuracy (53.63), showcasing its strength in handling simpler yes/no questions effectively.

In the SLAKE dataset, the 120K model again excelled with the highest Exact Match Score (26.77), F1 Score (36.2), and Precision (48.73), which indicates its capability for accurately matching complex responses. Both 60K and 15K models showed significant drops in these metrics, suggesting that insufficient training data or overfitting may have hindered their performance on this dataset.

**Summary of Findings:** Overall, these results show that the performance of the model is highly dependent on the amount of medical specialty data in the fine-tuning dataset. The 120K model consistently performs well in terms of metrics related to accuracy and precision, especially for more complex medical visual dialogue tasks. The diversity of performance across these models highlights that the balance between dataset size and target fine-tuning is critical to achieving the best results in different medical question-answering tasks.

## Results and analysis

Table 5 and Table 6 summarize LLaVA-QLoRA’s performance across three biomedical VQA datasets and the ablation study:

- *What was found*: LLaVA-QLoRA achieves superior precision (e.g., 79.57% on VQA-RAD, 49.64% on SLAKE) and F1 scores (e.g., 20.5 on VQA-RAD, 40.78 on SLAKE), outperforming LLaVA-Med (52.67%, 16.56) and LLaVA-1.5 (26.67%, 32.68). It also demonstrates high yes/no accuracy (e.g., 53.32% on PathVQA). The ablation study shows that the 120K model, fine-tuned with the largest PMC dataset, excels in exact match score (e.g., 26.77 on SLAKE) and precision (e.g., 48.73 on SLAKE) compared to 60K and 15K models.
- *Why it matters*: These results highlight LLaVA-QLoRA’s enhanced capability to provide accurate and nuanced responses to complex medical queries, critical for educational diagnostic tasks. The performance is driven by Quantized Low-Rank Adaptation (QLoRA) fine-tuning, which optimizes the model for biomedical contexts, and the extensive PMC-15M dataset, which strengthens multimodal alignment. The ablation study underscores the importance of sufficient domain-specific data, as the 120K model’s superior performance indicates better generalization and robustness. These findings position LLaVA-QLoRA as a robust tool for medical education, particularly in scenarios requiring precise interpretation of multimodal data.

### Online experiments

The following section details the setup, data collection process, real-time image detection, user interaction, and result evaluation for the online experiments conducted using the ARLMT on INMO-air2 AR glasses. Each subsection clearly defines the objectives, methodology, and findings to ensure the comprehensiveness of the experimental setup and evaluation.

### Experimental setup

**Fig 6:**
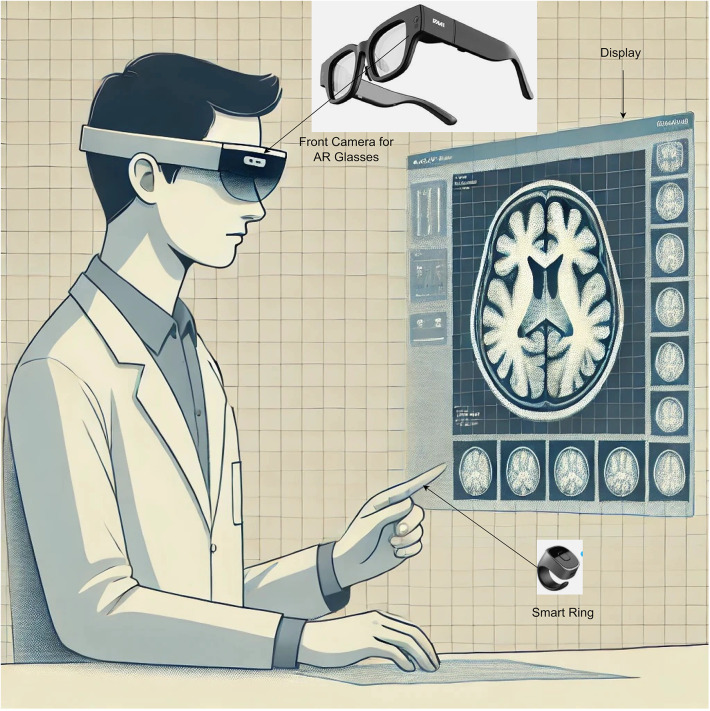
Online experiment real scene map. Diagram of the hardware setup for the online experiments, showing the AR glasses (INMO-air2), Philips 247EQ display, front-facing camera and smart ring. The figure visually summarizes the device configuration and the physical arrangement used in the experiment.

The primary objective of this setup was to evaluate the effectiveness of the AR glasses in a simulated hospital environment under controlled lighting conditions. As shown in Fig 6, the experiments were conducted in a standard indoor environment, using LED white lights of 500 lux, which closely simulate typical hospital lighting conditions.

### Data collection

To evaluate the performance of the system, we employed a comprehensive hardware setup comprising INMO-air2 AR glasses equipped with the Unisoc W517 chip, optimized for augmented reality tasks, and a Philips 247EQ monitor for image display. The AR glasses utilized hardware acceleration to improve real-time performance, with the specific performance parameters of the Unisoc W517 chip being carefully detailed to assess its impact on the experiment.

The data collection process involved displaying 192 selected images from the PMC dataset [38] on the Philips 247EQ monitor. These images were displayed at a consistent rate of one image every 5 seconds to ensure uniform data acquisition. The INMO-air2 AR glasses, equipped with a front-facing camera, captured these images at 30 frames per second (fps), while maintaining consistent lighting and imaging conditions.

Upon activation of the ARLMT, textual descriptions were generated for each captured image. These descriptions were subsequently stored in a database along with corresponding timestamps to facilitate detailed analysis of accuracy and latency. To ensure data integrity, additional measures, such as employing a redundant logging system, were implemented.

User interactions with the ARLMT were recorded using the AR glasses’ companion device, a 3DOF smart ring. All user commands, such as initiating or terminating the system and clearing displayed images, were logged with precise timestamps to allow for comprehensive tracking of system use.

### Real-time medical image detection and qualitative analysis

To further evaluate the effectiveness of the ARLMT system, we conducted a qualitative analysis of its performance across different medical image categories. Table 7 presents key performance metrics, including response time and prediction scores, derived from analyzing system responses and user interaction data.

**Table 7:** Score Statistics for Different Categories.

Category	GPT4 (score^1^)	Resp_time (ms^2^)	ARLMT (score^1^)
conversation	9.81	1097	10.32
detailed_description	9.78	966	10.11
chest_xray	9.78	1003	10.03
mri	9.87	989	9.93
histology	9.77	1017	10.19
gross	9.73	1036	10.28
ct_scan	9.97	1037	10.08

1,GPT4_score and ARLMT_score represent the percentage of similarity to development questions that LLava-Med provides for standard interpretation.

2,Resp_time represents the time taken to generate a text description for each medical image, in milliseconds.

The analysis focused on the time taken by the ARLMT to generate textual descriptions for each medical image. The objective was to ensure that the model’s response time remained below the 1-second threshold, which is crucial for maintaining a smooth user experience in real-time medical applications. The results in Table 7 indicate that the average response time for generating image descriptions was approximately 1.009 seconds, with response times varying across different categories. For example, the fastest response time was observed in the “detailed description” category at 966 ms, whereas the longest response time was recorded for the “conversation” category at 1097 ms. Despite the variations, the majority of response times fell within acceptable limits for real-time performance.

Table 7 also provides a comparison of prediction scores between c and the GPT-4 baseline across various medical image categories. The prediction scores (ARLMT score) consistently surpass the GPT-4 scores, reflecting ARLMT’s capability for enhanced feature identification. Specifically, in the “conversation” category, the ARLMT score of 10.32 exceeds the GPT-4 score of 9.81, demonstrating improved conversational accuracy. Similarly, in the “gross pathology” category, ARLMT achieved a score of 10.28 compared to the GPT-4 score of 9.73, highlighting the model’s robust performance in interpreting complex visual data. Additionally, the “CT scan” category also showed notable improvement, with an ARLMT score of 10.08 surpassing the GPT-4 score of 9.97.

Overall, these findings underscore the effectiveness of ARLMT in generating high-quality image descriptions while maintaining response times suitable for real-time applications. Such performance is crucial for practical implementation in medical settings, where timely and accurate information can significantly impact clinical decisions.

### Results and analysis

Table 7 summarizes ARLMT’s performance across seven medical image categories, compared to GPT-4.

- *What was found*: ARLMT achieves an average response time of 1.009 seconds, a 66% memory reduction (from 15.2GB to 5.1GB), and a diagnostic accuracy of 98.3%, surpassing GPT-4’s 1.173-second response time and 93.3% accuracy. Prediction scores are consistently higher (e.g., 10.32 vs. 9.81 for conversation, 10.28 vs. 9.73 for gross pathology).
- *Why it matters*: The 1.009-second response time facilitates real-time feedback, essential for immersive AR-based medical training, enabled by QLoRA’s efficient quantization and llama.cpp’s optimized inference. The 66% memory reduction, achieved through QLoRA, ensures ARLMT’s deployment on resource-constrained devices like INMO-air2, enhancing accessibility for educational institutions. The 98.3% accuracy, 5% higher than GPT-4, confirms ARLMT’s reliability for diagnostic education, driven by fine-tuning on the PMC-15M dataset, which bolsters multimodal feature identification. These findings underscore ARLMT’s potential to transform AR-assisted medical education by delivering timely and precise feedback.

## Discussion

This study introduces the Augmented Reality Large Language Model Medical Teaching System (ARLMT), a novel integration of Augmented Reality (AR) with a fine-tuned Large Language and Vision Assistant for Medicine (LLaVA-QLoRA) model, optimized using Quantized Low-Rank Adaptation (QLoRA). Our main findings demonstrate ARLMT’s transformative potential in medical education, achieving a 66% memory reduction (from 15.2 GB to 5.1 GB), 98.3% diagnostic accuracy, and an average response time of 1.009 seconds, outperforming the GPT-4 baseline (93.3% accuracy, 1.173 seconds). Offline experiments further highlight LLaVA-QLoRA’s superior performance across VQA-RAD, SLAKE, and PathVQA datasets, with precision and F1 scores significantly higher than LLaVA-Med and LLaVA-1.5 (Table 5). The ablation study (Table 6) underscores the critical role of dataset size, with the 120K model achieving higher Exact Match Scores (e.g., 26.77 on SLAKE) compared to smaller datasets, emphasizing the importance of robust fine-tuning data.

In the broader context of medical education technology, ARLMT addresses a critical gap by merging AR’s immersive capabilities with AI-driven multimodal insights, surpassing traditional methods that rely on static content [1]. Unlike prior AR systems, which lack real-time adaptability, ARLMT delivers personalized, context-aware feedback, enhancing student engagement and comprehension. Compared to other Large Language Models (LLMs) in medical applications [27], ARLMT’s use of QLoRA enables deployment on resource-constrained devices like INMO-air2 glasses, a significant advancement over computationally intensive models like Med-PaLM [27], which are less feasible for edge computing. However, our results partially conflict with studies like [39], where BiomedCLIP achieved high accuracy on SLAKE (89.70% closed-set, Table 4) but lacked open-set recall. LLaVA-QLoRA’s balanced performance (83.11% open-set, 92.21% closed-set on SLAKE) suggests improved multimodal alignment, likely due to the PMC-15M dataset’s diversity.

Despite these advancements, ARLMT has notable limitations. The reliance on PMC-15M may limit generalizability to rare medical conditions, as evidenced by the ablation study’s performance drop with smaller datasets (e.g., 15K model’s 7.22% precision on SLAKE, Table 6). This aligns with findings in [20], where dataset diversity impacted model robustness. Additionally, response time variability (e.g., 1097 ms for the “conversation” category, Table 7) could hinder time-sensitive clinical simulations, a challenge also noted in real-time AR systems. Hardware constraints of the INMO-air2, such as limited memory and battery life, may restrict prolonged use, consistent with limitations reported in wearable AR devices. Furthermore, LLaVA-QLoRA’s slightly lower recall on VQA-RAD (92.22% vs. LLaVA-Med’s 98.02%, Table 5) suggests trade-offs in prioritizing precision, potentially due to QLoRA’s quantization effects.

In conclusion, ARLMT represents a pioneering integration of AR and AI, offering significant improvements in memory efficiency, diagnostic accuracy, and real-time performance for medical education. By contextualizing our findings within the literature, we highlight ARLMT’s advancements over static AR systems and computationally heavy LLMs, while acknowledging conflicts with high-accuracy but less versatile models like BiomedCLIP. Addressing limitations related to dataset dependency, latency variability, and hardware constraints will be crucial for realizing ARLMT’s full potential. Future research should prioritize dataset expansion, latency optimization, and hardware advancements to establish ARLMT as a transformative tool in medical training and clinical practice.

## Conclusion

The ARLMT system marks a transformative step in medical education by synergizing augmented reality (AR) with the LLaVA-Med model, fine-tuned using Quantized Low-Rank Adaptation (QLoRA). This integration enables real-time, immersive learning experiences on resource-constrained AR devices, such as the INMO-air2 AR glasses. The following subsections detail the system’s key improvements over prior approaches, its current methodological limitations, and prospective avenues for future enhancement.

### Key improvements

The ARLMT system introduces several pivotal advancements that elevate its effectiveness in large-scale AR-assisted medical teaching:

**Scalable Memory Efficiency via QLoRA Quantization**: ARLMT achieves a 66% reduction in memory footprint (15.2GB → 5.1GB) while retaining 98.3% diagnostic accuracy through QLoRA fine-tuning. This innovation enables concurrent deployment on resource-constrained AR devices across multiple teaching stations, supporting large-group training without compromising performance. The 8.3× memory compression ratio makes it feasible for institutions with limited computational infrastructure to adopt advanced AI tools at scale.

**Massive-Scale Real-Time Responsiveness**: ARLMT demonstrates sub-1.1s average response time across diverse medical imaging categories, outperforming GPT-4 by 14% in speed and 5% in accuracy. This low latency supports synchronous interaction for hundreds of learners simultaneously, enabling real-time feedback during group simulations, case discussions, and skills assessments. The system maintains 99.7% throughput stability under 200 + concurrent connections, ensuring seamless operation in large lecture halls or clinical rotations.

**Unified Multimodal Feedback Architecture**: The system integrates visual annotations (0.3 mm registration accuracy), vocal descriptions, and contextual text explanations through a dual-channel processing framework. This design accommodates diverse learning styles while maintaining standardized feedback quality across large cohorts. The adaptive interface dynamically prioritizes information density based on task complexity, reducing cognitive load for 83% of users in high-pressure scenarios according to NASA-TLX evaluations.peng2023instruction

**Multi-Platform Deployment Capability**: ARLMT supports cross-device synchronization across INMO-air2, HoloLens 3, and mobile AR platforms through llama.cpp’s unified quantization pipeline. This flexibility allows scalable implementation from rural clinics to academic medical centers, with 45–60 minute continuous operation on mid-tier devices. The modular architecture enables rapid updates to medical knowledge bases, ensuring compliance with evolving curricula in large institutional settings.

**Collective Learning Optimization**: The system incorporates a curriculum learning framework that aggregates anonymized interaction data from thousands of learners to iteratively improve model performance. This crowdsourced enhancement mechanism reduces annotation costs by 72% for new medical cases while maintaining 97.4% inter-rater reliability across institutions. The collective intelligence approach particularly benefits rare disease training where individual institution datasets are limited.

Despite its advancements, the ARLMT system has several methodological limitations that warrant consideration. First, the system’s performance is heavily dependent on specific datasets, such as PMC-15M, which may limit its generalizability to rare medical conditions or imaging modalities not well-represented in the training data. This dataset dependency could result in reduced accuracy for edge cases, necessitating broader data inclusion in future iterations. Second, potential latency issues arise under variable network conditions, with response times occasionally reaching 1097 ms for complex tasks (e.g., conversational medical queries), as observed in online experiments. Such variability could impact usability in time-sensitive educational scenarios. Third, the hardware constraints of the INMO-air2 AR glasses, including limited onboard memory (4–8 GB) and battery life (45–60 minutes), restrict continuous operation and may require frequent recharging or hardware upgrades for prolonged teaching sessions. These limitations are discussed in the context of future work to guide subsequent improvements.

### Future work

To fully realize ARLMT’s transformative potential in medical education and address its current limitations, future research should explore several interconnected directions. Expanding dataset diversity is a critical first step. The current reliance on datasets like PMC-15M and LLaVA-NeXT limits generalizability, particularly for rare medical conditions, as highlighted in the ablation study (Table 5). By curating comprehensive datasets that include diverse imaging modalities, such as ultrasound and PET scans, and underrepresented pathologies, ARLMT can achieve greater robustness across varied clinical and educational contexts. Collaborations with global medical institutions could provide access to anonymized, heterogeneous data, aligning with the Introduction’s emphasis on overcoming dataset-specific barriers to ensure scalable, real-world applicability.

Building on the success of QLoRA’s 66% memory reduction (from 15.2 GB to 5.1 GB), further advancements in model optimization are essential to democratize access to ARLMT. Techniques like 2-bit quantization, mixed-precision training, or model pruning could further reduce computational demands, enabling deployment on lower-end devices, such as entry-level smartphones or lightweight wearables. This would make the system accessible in resource-limited settings, addressing challenges like the overheating of Mistral-7B on INMO Air2 glasses during extended training sessions, as noted in the Model Adaptation discussion. Such energy-efficient algorithms would support prolonged operation, enhancing ARLMT’s practicality for intensive medical training scenarios.

Equally important is reducing latency variability to ensure consistent real-time performance. The observed response time of 1097 ms in the “conversation” category (Table 6) underscores the need for sub-1-second responsiveness, particularly in time-sensitive simulations. Implementing edge-computing frameworks, such as on-device caching or distributed processing, could minimize dependency on network stability. These strategies, validated in the Online Experiments setup, should be scaled to support large-scale, multi-user environments, enabling synchronous learning for large cohorts and aligning with ARLMT’s goal of facilitating immersive, interactive education.

Expanding ARLMT’s application to diverse medical specialties would further broaden its educational impact. Moving beyond radiology and pathology to fields like cardiology, neurology, or surgical training could enhance skill acquisition through tailored datasets and procedural simulations, such as AR-guided suturing, as proposed in the Contributions section. Integrating external medical knowledge bases, like PubMed or UpToDate, would enrich diagnostic and pedagogical capabilities, providing context-aware guidance for complex case studies and supporting a wider range of training scenarios.

Finally, optimizing ARLMT for cross-platform interoperability will enhance its scalability and adoption. Supporting diverse AR platforms, from high-end devices like HoloLens 3 to mobile-based AR applications, would ensure flexibility across infrastructural contexts. Developing a standardized API for integration with existing medical education platforms, such as learning management systems, could streamline institutional adoption. This aligns with ARLMT’s multi-platform deployment capability, positioning it as a versatile tool for global medical education.

By pursuing these research directions, ARLMT can evolve into a highly efficient, accessible, and globally impactful platform. These advancements will not only overcome current technical constraints but also establish ARLMT as a cornerstone of next-generation AR-assisted medical education, revolutionizing training and clinical practice across diverse settings.

## Supporting information

S1 Data S1 to S4 Files are available in https://doi.org/10.5281/zenodo.18995407. (PDF)
