# Supplementary material for: Research on the application of LLaVA model based on QLoRA fine-tuning in medical teaching
Source: PLoS One. 2026 Jul 14;21(7):e0328408. doi: 10.1371/journal.pone.0328408 (PMC13367897; doi:10.1371/journal.pone.0328408)
Supplement: S1 Data — (PDF) [file pone.0328408.s001.pdf]

Supporting Information Captions

S1 File. S1 File. This 7z archive (split into multiple parts due to file size limit) includes JSON files for the first-step medical concept alignment and the second-step visual capability training of the LLaVA-Med model. The file is available at DOI: `https://doi.org/10.5281/zenodo.18995407`. All parts can be merged and decompressed using the free 7-Zip software.

S2 File. S2 File. This 7z archive contains the project code for the ARLMT system. The file is available at DOI: `https://doi.org/10.5281/zenodo.18995407`. All parts can be merged and decompressed using the free 7-Zip software.

S3 File. S3 File. This 7z archive (split into multiple parts due to file size limit) includes the fine-tuned and quantized model files for the LLaVA-Med model, optimized for efficient deployment on resource-constrained devices. The file is available at DOI: `https://doi.org/10.5281/zenodo.18995407`. All parts can be merged and decompressed using the free 7-Zip software.

S4 File. S4 File. This 7z archive contains a PDF file with a numbered hierarchical table of contents for the manuscript, listing all sections, subsections, and subsubsections to improve navigation and address organizational feedback. The file is available at DOI: `https://doi.org/10.5281/zenodo.18995407`. All parts can be merged and decompressed using the free 7-Zip software.
