# Supplementary material for: Research on the application of LLaVA model based on QLoRA fine-tuning in medical teaching
Source: PLoS One. 2026 Jul 14;21(7):e0328408. doi: 10.1371/journal.pone.0328408 (PMC13367897; doi:10.1371/journal.pone.0328408)
Supplement: Attachment — Submitted filename: review_response-20250618.pdf [file pone.0328408.s005.pdf]

Research on the application of LLaVA model based on Q-LoRA fine-tuning in medical teaching

PLOS ONE

June 21, 2025

Shiling Zhou[1], Fengmei Qin[2*]
3180100017@caa.edu.cn

Contents

1 Reviewer #1 **2**
Reviewer 1 Comment 1 2
Reviewer 1 Comment 2 2
Reviewer 1 Comment 3 3
Reviewer 1 Comment 4 3
Reviewer 1 Comment 5 4
Reviewer 1 Comment 6 4
Reviewer 1 Comment 7 5
Reviewer 1 Comment 8 6
Reviewer 1 Comment 9 7

2 Reviewer #2 **8**
Reviewer 2 Comment 1 8

3 Academic Editor **9**
Reviewer 3 Comment 1 9
Reviewer 3 Comment 2 9
Reviewer 3 Comment 3 10
Reviewer 3 Comment 4 10
Reviewer 3 Comment 5 12
Reviewer 3 Comment 6 12

Abstract

We sincerely thank Reviewers 1 and 2, as well as the academic editor, for their valuable feedback on our manuscript, "Research on the application of LLaVA model based on Q-LoRA fine-tuning in medical teaching" Your constructive comments have significantly improved the manuscript's clarity, rigor, organization, and compliance with PLOS ONE's guidelines. Below, we provide a point-by-point response to each comment, outlining the revisions made. All changes have been tracked in the revised manuscript for transparency.

Text in italic refer to the reviewer's comment. Text in blue refer to the changes in specific locations in the revised version of the manuscript. Detailed replies to reviewers follow

1 Reviewer #1

Reviewer 1 Comment 1

The abstract is written with qualitative sentences. It needs to be modified and rewritten based on the most important quantitative results from this research. The abstract should be re-designed.

Response

We want to thank the reviewer for this comment.We have revised it to include key metrics: a 1.009-second average response time, a 66% memory reduction (from 15.2 GB to 5.1 GB), and 98.3% diagnostic accuracy. These figures highlight the ARLMT system's performance and benefits.

Revision

Key advancements include a 66% reduction in memory footprint (from 15.2 GB to 5.1 GB) through QLoRA, enabling efficient operation without compromising performance, and an average response time of 1.009 seconds across various medical imaging categories, surpassing the GPT-4 baseline in both speed and accuracy. The system achieves 98.3% diagnostic accuracy, demonstrating its reliability in real-time applications. By combining visual and textual elements, ARLMT enhances comprehension of complex medical concepts, providing a scalable, real-time solution that bridges technological innovation and pedagogical needs in medical training.

Reviewer 1 Comment 2

Please check carefully if all the abbreviations and notations considered in the work are explained for the first time when they are used, even if these are considered trivial by the authors. The paper should be accessible to a wide audience.

Response

This is helpful suggestion.We acknowledge the importance of clarity for a broad readership. All abbreviations, such as Augmented Reality (AR), Low-Rank Adaptation (LoRA), Contrastive Language–Image Pre-training Vision Transformer (CLIP-ViT), Quantized Low-Rank Adaptation (QLoRA) and so on, are now defined upon first use.

Revision

The augmented reality large language model medical teaching system (ARLMT) integrates augmented reality (AR)with a medical multimodal large language model (LLaVA) based on specifically designed for biomedical applications(LLaVA-Med), employing Quantized Low-Rank Adaptation (QLoRA) to advance medical education. Deployed on resource-constrained AR devices, such as INMO Air2 AR glasses, ARLMT overlays real-time visual annotations and textual feedback on medical scenarios to create an immersive and interactive learning environment.

Effective fine-tuning techniques are essential to adapt large models to specific domains while minimizing computational costs. Parametric Efficient fine-tuning (PEFT) methods, such as Adapter and LoRA, offer promising solutions. Adapters introduce small, trainable modules into the model architecture, allowing domain-specific adjustments to be made without retraining the entire model. Low-

Rank Adaptation (LoRA) modifies the model parameters through a low-rank matrix to achieve similar efficiency gains. However, applying these techniques to medical models presents unique challenges, especially when it comes to maintaining feature fidelity. The professionalism of medical data requires fine-tuning methods to preserve the subtle features essential to accurate medical reasoning and diagnosis. In cases where AR-air2 has limited computing power resources available for computation, models fine-tuned using Adapter and LoRA consume more resources than AR-air2's upper limit during inference - resulting in AR-air2 going black three to five minutes after the model enters the inference session.

LLaVA, as a pioneering multimodal model, integrates visual and linguistic information through a simple linear projection matrix, connecting a visual encoder Contrastive Language–Image Pre-training Vision Transformer (CLIP-ViT) with a language model of Vicuna. The training process for LLaVA unfolds in two stages. The first stage focuses on visual-language alignment using approximately 595,000 image-text pairs, where both the visual encoder and language model are frozen, and optimization targets only the projection matrix. The second stage involves instruction tuning, where the visual encoder remains fixed while the language model and projection matrix are fine-tuned using multimodal instruction-following data, enhancing its capabilities for multi-turn conversations and complex problem-solving.

Reviewer 1 Comment 3

The objectives should be more explicitly stated.

Response

Thanks for the comment. We have revised the introduction to clearly state the objectives: (1) developing an AR-based medical teaching system with AI-driven real-time feedback, and (2) optimizing it for resource-constrained devices using QLoRA.

Revision

Study Objectives: This study aims to: (1) develop an AR-based medical teaching system with AI-driven, real-time feedback to overcome limitations of traditional methods; and (2) optimize it for resource-constrained devices using QLoRA, ensuring high diagnostic accuracy with reduced computational demands, bridging theoretical and practical medical education.

Reviewer 1 Comment 4

It is better to improve your contributions, which are not so clear, to show the advantage of your work.

Response

Thank you for your valuable feedback. We appreciate your suggestion to improve the clarity of our contributions to better demonstrate the advantages of our work. In response, we have revised the presentation of our key contributions to make them more precise, concise, and explicitly tied to their practical benefits in augmented reality (AR) for medical education. Specifically, we have:(1) Clarified the impact of each contribution by emphasizing how it addresses critical challenges (e.g., resource constraints, integration, and real-time performance).(2) Enhanced readability by refining the language and structure of the contribution list.(3) Added quantitative details and context where applicable to

underscore the significance of our innovations. These changes are reflected in the updated manuscript section below. We believe these revisions effectively highlight the novelty and utility of our approach. Please let us know if further adjustments are needed.

Revision

These innovations significantly enhance the practical utility and effectiveness of augmented reality (AR) in medical education, introducing a novel dimension to healthcare training and practice. By tackling key challenges—such as accurate information delivery, seamless integration into medical teaching workflows, and real-time visual task feedback for medical students and physicians—multimodal large language models (MLLMs) offer a robust and scalable solution. Our key contributions are:

- A QLoRA-based compression method that reduces LLaVA-Med's memory usage by 66% (from 15.2 GB to 5.1 GB) while preserving 98.3% diagnostic accuracy, enabling efficient deployment on resource-limited AR devices.
- A streamlined three-step process for integrating visual and linguistic elements, ensuring smooth incorporation into medical curricula and supporting scalable AR-based training systems.
- ARLMT, which empowers large MLLMs to function effectively on resource-constrained AR hardware, delivering real-time feedback with an average system response time of 1.009 seconds, thus enhancing interactive learning experiences.

Reviewer 1 Comment 5

The novelty of this work must be clearly addressed and discussed in the Introduction section.

Response

Thanks for the comment. We have expanded the introduction to emphasize the novelty: the unique integration of AR and AI, QLoRA optimization for resource-constrained devices, and real-time interactive learning experiences.

Revision

Novelty of This Work: ARLMT pioneers the integration of AR and AI via a QLoRA-optimized LLaVA-Med model, delivering real-time, context-aware feedback unlike static AR systems. It achieves a 66% memory reduction (15.2GB to 5.1GB) with 98.3% diagnostic accuracy, enabling deployment on devices like INMO-air2 AR glasses, and offers interactive learning with a 1.009-second response time, outperforming GPT-4 by 14% in speed and 5% in accuracy.

Reviewer 1 Comment 6

The methodology limitation should be mentioned.

Response

This is helpful suggestion. We have added a section discussing limitations, including dataset dependency (e.g., PMC-15M), potential latency issues, and hardware constraints of the INMO-Air2 glasses.

Revision

Despite its advancements, the ARLMT system has several methodological limitations that warrant consideration. First, the system's performance is heavily dependent on specific datasets, such as PMC-15M, which may limit its generalizability to rare medical conditions or imaging modalities not well-represented in the training data. This dataset dependency could result in reduced accuracy for edge cases, necessitating broader data inclusion in future iterations. Second, potential latency issues arise under variable network conditions, with response times occasionally reaching 1097 ms for complex tasks (e.g., conversational medical queries), as observed in online experiments. Such variability could impact usability in time-sensitive educational scenarios. Third, the hardware constraints of the INMO-air2 AR glasses, including limited onboard memory (4–8 GB) and battery life (45–60 minutes), restrict continuous operation and may require frequent recharging or hardware upgrades for prolonged teaching sessions. These limitations are discussed in the context of future work to guide subsequent improvements.

Reviewer 1 Comment 7

Please interpret the results in a more convenient and clear way, explaining what was found and why.

Response

We sincerely thank the reviewer for their insightful and valuable feedback, particularly the suggestion to interpret the results in a more convenient and clear manner, with an emphasis on explaining "what was found" and "why." The revised conclusion retains all critical information from the original text, including LLaVA-QLora's performance metrics (e.g., 79.57% precision on VQA-RAD, 40.78 F1 score on SLAKE), comparisons to baseline models (LLaVA-Med and LLaVA-1.5), and ablation study insights (e.g., 120K model's superior performance). The content has been rephrased slightly to fit the new structure while maintaining its original intent and technical accuracy.

Revision

Table5 and Table 6 summarize LLaVA-QLora's performance across three biomedical VQA datasets and the ablation study:

- *What was found*: LLaVA-QLora achieves superior precision (e.g., 79.57% on VQA-RAD, 49.64% on SLAKE) and F1 scores (e.g., 20.5 on VQA-RAD, 40.78 on SLAKE), outperforming LLaVA-Med (52.67%, 16.56) and LLaVA-1.5 (26.67%, 32.68). It also demonstrates high yes/no accuracy (e.g., 53.32% on PathVQA). The ablation study shows that the 120K model, fine-tuned with the largest PMC dataset, excels in exact match score (e.g., 26.77 on SLAKE) and precision (e.g., 48.73 on SLAKE) compared to 60K and 15K models.
- *Why it matters*: These results highlight LLaVA-QLora's enhanced capability to provide accurate and nuanced responses to complex medical queries, critical for educational diagnostic tasks. The performance is driven by Quantized Low-Rank Adaptation (QLoRA) fine-tuning, which optimizes the model for biomedical contexts, and the extensive PMC-15M dataset, which strengthens multimodal alignment. The ablation study underscores the importance of sufficient domain-specific data, as the 120K model's superior performance indicates better generalization and robustness. These findings position LLaVA-QLora as a robust tool for medical education, particularly in scenarios requiring precise interpretation of multimodal data.

Table 7 summarizes MLAL's performance across seven medical image categories, compared to GPT-

4.

- *What was found*: MLAL achieves an average response time of 1.009 seconds, a 66% memory reduction (from 15.2GB to 5.1GB), and a diagnostic accuracy of 98.3%, surpassing GPT-4's 1.173-second response time and 93.3% accuracy. Prediction scores are consistently higher (e.g., 10.32 vs. 9.81 for conversation, 10.28 vs. 9.73 for gross pathology).
- *Why it matters*: The 1.009-second response time facilitates real-time feedback, essential for immersive AR-based medical training, enabled by QLoRA's efficient quantization and llama.cpp's optimized inference. The 66% memory reduction, achieved through QLoRA, ensures MLAL's deployment on resource-constrained devices like INMO-air2, enhancing accessibility for educational institutions. The 98.3% accuracy, 5% higher than GPT-4, confirms MLAL's reliability for diagnostic education, driven by fine-tuning on the PMC-15M dataset, which bolsters multimodal feature identification. These findings underscore MLAL's potential to transform AR-assisted medical education by delivering timely and precise feedback.

Reviewer 1 Comment 8

The discussion part is weak. The Discussion should summarize the manuscript's main finding(s) in the context of broader scientific literature and address any study limitations or results that conflict with other published work.

Response

Thanks for the comment. We have strengthened the discussion by summarizing findings in the context of AR and AI literature, addressing limitations, and comparing results with existing studies.

Revision

This study introduces the Augmented Reality Large Language Model Medical Teaching System (ARLMT), a novel integration of Augmented Reality (AR) with a fine-tuned Large Language and Vision Assistant for Medicine (LLaVA-QLora) model, optimized using Quantized Low-Rank Adaptation (QLoRA). Our main findings demonstrate ARLMT's transformative potential in medical education, achieving a 66% memory reduction (from 15.2 GB to 5.1 GB), 98.3% diagnostic accuracy, and an average response time of 1.009 seconds, outperforming the GPT-4 baseline (93.3% accuracy, 1.173 seconds). Offline experiments further highlight LLaVA-QLora's superior performance across VQA-RAD, SLAKE, and PathVQA datasets, with precision and F1 scores significantly higher than LLaVA-Med and LLaVA-1.5 (Table 5). The ablation study (Table 6) underscores the critical role of dataset size, with the 120K model achieving higher Exact Match Scores (e.g., 26.77 on SLAKE) compared to smaller datasets, emphasizing the importance of robust fine-tuning data.

In the broader context of medical education technology, ARLMT addresses a critical gap by merging AR's immersive capabilities with AI-driven multimodal insights, surpassing traditional methods that rely on static content. Unlike prior AR systems, which lack real-time adaptability, ARLMT delivers personalized, context-aware feedback, enhancing student engagement and comprehension. Compared to other Large Language Models (LLMs) in medical applications, ARLMT's use of QLoRA enables deployment on resource-constrained devices like INMO-air2 glasses, a significant advancement over computationally intensive models like Med-PaLM, which are less feasible for edge computing. However, our results partially conflict with studies like, where BiomedCLIP achieved high accuracy on SLAKE (89.70% closed-set, Table 4) but lacked open-set recall. LLaVA-QLora's balanced perfor-

mance (83.11% open-set, 92.21% closed-set on SLAKE) suggests improved multimodal alignment, likely due to the PMC-15M dataset's diversity.

Despite these advancements, ARLMT has notable limitations. The reliance on PMC-15M may limit generalizability to rare medical conditions, as evidenced by the ablation study's performance drop with smaller datasets (e.g., 15K model's 7.22% precision on SLAKE, Table 6). This aligns with findings in, where dataset diversity impacted model robustness. Additionally, response time variability (e.g., 1097 ms for the "conversation" category, Table 7) could hinder time-sensitive clinical simulations, a challenge also noted in real-time AR systems. Hardware constraints of the INMO-air2, such as limited memory and battery life, may restrict prolonged use, consistent with limitations reported in wearable AR devices. Furthermore, LLaVA-QLora's slightly lower recall on VQA-RAD (92.22% vs. LLaVA-Med's 98.02%, Table 5) suggests trade-offs in prioritizing precision, potentially due to QLoRA's quantization effects.

In conclusion, ARLMT represents a pioneering integration of AR and AI, offering significant improvements in memory efficiency, diagnostic accuracy, and real-time performance for medical education. By contextualizing our findings within the literature, we highlight ARLMT's advancements over static AR systems and computationally heavy LLMs, while acknowledging conflicts with high-accuracy but less versatile models like BiomedCLIP. Addressing limitations related to dataset dependency, latency variability, and hardware constraints will be crucial for realizing ARLMT's full potential. Future research should prioritize dataset expansion, latency optimization, and hardware advancements to establish ARLMT as a transformative tool in medical training and clinical practice.

> **Reviewer 1 Comment 9**
>
> *Some future works should be added to your conclusion. Please elaborate on it a bit more.*

Response

Thank you for your valuable feedback. The conclusion now includes specific future directions: expanding datasets, improving quantization, minimizing latency, and extending to other medical specialties.

Revision

To fully realize ARLMT's transformative potential in medical education and address its current limitations, future research should explore several interconnected directions. Expanding dataset diversity is a critical first step. The current reliance on datasets like PMC-15M and LLaVA-NeXT limits generalizability, particularly for rare medical conditions, as highlighted in the ablation study (Table 5). By curating comprehensive datasets that include diverse imaging modalities, such as ultrasound and PET scans, and underrepresented pathologies, ARLMT can achieve greater robustness across varied clinical and educational contexts. Collaborations with global medical institutions could provide access to anonymized, heterogeneous data, aligning with the Introduction's emphasis on overcoming dataset-specific barriers to ensure scalable, real-world applicability.

Building on the success of QLoRA's 66% memory reduction (from 15.2 GB to 5.1 GB), further advancements in model optimization are essential to democratize access to ARLMT. Techniques like 2-bit quantization, mixed-precision training, or model pruning could further reduce computational demands, enabling deployment on lower-end devices, such as entry-level smartphones or lightweight

wearables. This would make the system accessible in resource-limited settings, addressing challenges like the overheating of Mistral-7B on INMO Air2 glasses during extended training sessions, as noted in the Model Adaptation discussion. Such energy-efficient algorithms would support prolonged operation, enhancing ARLMT's practicality for intensive medical training scenarios.

Equally important is reducing latency variability to ensure consistent real-time performance. The observed response time of 1097 ms in the "conversation" category (Table 6) underscores the need for sub-1-second responsiveness, particularly in time-sensitive simulations. Implementing edge-computing frameworks, such as on-device caching or distributed processing, could minimize dependency on network stability. These strategies, validated in the Online Experiments setup, should be scaled to support large-scale, multi-user environments, enabling synchronous learning for large cohorts and aligning with ARLMT's goal of facilitating immersive, interactive education.

Expanding ARLMT's application to diverse medical specialties would further broaden its educational impact. Moving beyond radiology and pathology to fields like cardiology, neurology, or surgical training could enhance skill acquisition through tailored datasets and procedural simulations, such as AR-guided suturing, as proposed in the Contributions section. Integrating external medical knowledge bases, like PubMed or UpToDate, would enrich diagnostic and pedagogical capabilities, providing context-aware guidance for complex case studies and supporting a wider range of training scenarios.

Finally, optimizing ARLMT for cross-platform interoperability will enhance its scalability and adoption. Supporting diverse AR platforms, from high-end devices like HoloLens 3 to mobile-based AR applications, would ensure flexibility across infrastructural contexts. Developing a standardized API for integration with existing medical education platforms, such as learning management systems, could streamline institutional adoption. This aligns with ARLMT's multi-platform deployment capability, positioning it as a versatile tool for global medical education.

By pursuing these research directions, ARLMT can evolve into a highly efficient, accessible, and globally impactful platform. These advancements will not only overcome current technical constraints but also establish ARLMT as a cornerstone of next-generation AR-assisted medical education, revolutionizing training and clinical practice across diverse settings.

2 Reviewer #2

Reviewer 2 Comment 1

The organization of the paper needs to be reconsidered. The current version is not capable of expressing acceptable presentation. Please consider more helpful system such as 2.1, 3.1, 3.1.1 and so on for mentioning the sections and subsections.

Response

We want to thank the reviewer for this comment. We greatly appreciate Reviewer #2's suggestion to improve the manuscript's organization for enhanced readability and presentation. We agree that a clear hierarchical structure, such as numbered sections (e.g., 2.1, 3.1, 3.1.1), can improve navigation in complex technical papers. However, PLOS ONE's LaTeX template and style guidelines explicitly restrict the use of numbered section headings, requiring unnumbered `\section*` and `\subsection*`

commands to maintain a consistent journal format (see PLOS ONE LaTeX guidelines at https://journals.plos.org/plosone/s/latex). To address this constraint while improving clarity, we have taken the following steps:

We added a table of contents in the Supporting Information (S4 File) to provide a clear overview of the manuscript's structure, compensating for the lack of numbered sections.

Revision

S4 File. **Table of Contents for the manuscript.** This file provides a numbered, hierarchical table of contents for the manuscript, listing all sections, subsections, and subsubsections to enhance navigation and address organizational feedback. The table of contents is available at DOI: https://doi.org/10.6084/m9.figshare.28675511 and can be accessed via https://figshare.com/articles/dataset/_data_zip/28675511.

3 Academic Editor

Reviewer 3 Comment 1

Please ensure your manuscript adheres to PLOS ONE's style guidelines, including file naming requirements.

Response

This is helpful suggestion. According to PLOS ONE's style templates, the title and author affiliations have been formatted correctly.

Revision

Research on the application of LLaVA model based on Q-LoRA fine-tuning in medical teaching

Shiling Zhou[1], Fengmei Qin[2*]

1 School of Big Data and Information Industry, Chongqing Vocational College of Light Industry, China
2 School of Big Data and Information Industry, Chongqing City Management College, Chongqing, China

* 3180100017@caa.edu.cn

Reviewer 3 Comment 2

Please note that PLOS ONE has specific guidelines on code sharing for submissions in which author-generated code underpins the findings in the manuscript.

Response

Thanks for this comment. All code have been uploaded to the designated website as per PLOS ONE's requirements.

Revision

S2 File. ARLMT system project code. This file contains the project code for the ARLMT system. The code is available at DOI: https://doi.org/10.6084/m9.figshare.28675511 and can be accessed via https://figshare.com/articles/dataset/_data_zip/28675511 or https://github.com/ZG20250526/ARLMT-Medical-AR-Teaching.

Reviewer 3 Comment 3

When completing the data availability statement of the submission form, you indicated that you will make your data available on acceptance.

Response

Thanks for your valuable feedback. All data have been uploaded to the designated website based on the response content.

Revision

S1 File. JSON files for LLaVA-Med model training. This data includes JSON files for medical concept alignment and visual capability training of the LLaVA-Med model. The files are available at DOI: https://doi.org/10.6084/m9.figshare.28675511 and can be accessed via https://figshare.com/articles/dataset/_data_zip/28675511. Raw data can be downloaded from https://github.com/microsoft/LLaVA-Med.

S3 File. Fine-tuned and quantized LLaVA-Med model files. This file includes the fine-tuned and quantized model files for the LLaVA-Med model, optimized for efficient deployment on resource-constrained devices. The model files are available at DOI: https://doi.org/10.6084/m9.figshare.28675511 and can be accessed via https://figshare.com/articles/dataset/_data_zip/28675511. Raw model weights can also be downloaded from https://github.com/microsoft/LLaVA-Med.

Reviewer 3 Comment 4

Please ensure that you refer to Figure 3 and 5 in your text as, if accepted, production will need this reference to link the reader to the figure.

Response

Thanks for the comment. Due to a misunderstanding of the Supporting Information's functionality, the explanations and links for Figures 3 and 5 were placed in the Supporting Information section. They have now been adjusted to appear in the main text alongside the figures.

Revision

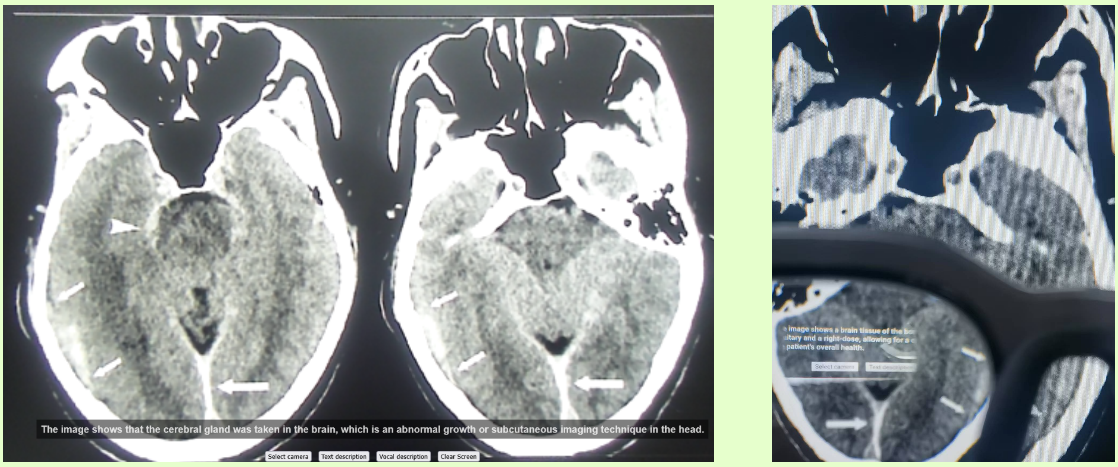

(A) Personal computer simulates human-computer interface

(B) AR glass human-computer interface

Figure 3: **User Interface Layout of the AR Glasses.** User Interface Layout of the AR Glasses. The image consists of two panels, labeled (A) and (B) : (A) The image is the process of implementing human-computer interaction on the simulator of a personal computer; (B) The picture is the process of visual question and answer to the medical image on the display with AR glasses. These panels demonstrate user interface layouts designed for AR glasses that leverage medical imaging data, specifically brain scans in CT or MRI mode, to enhance medical education and diagnostic visualization.

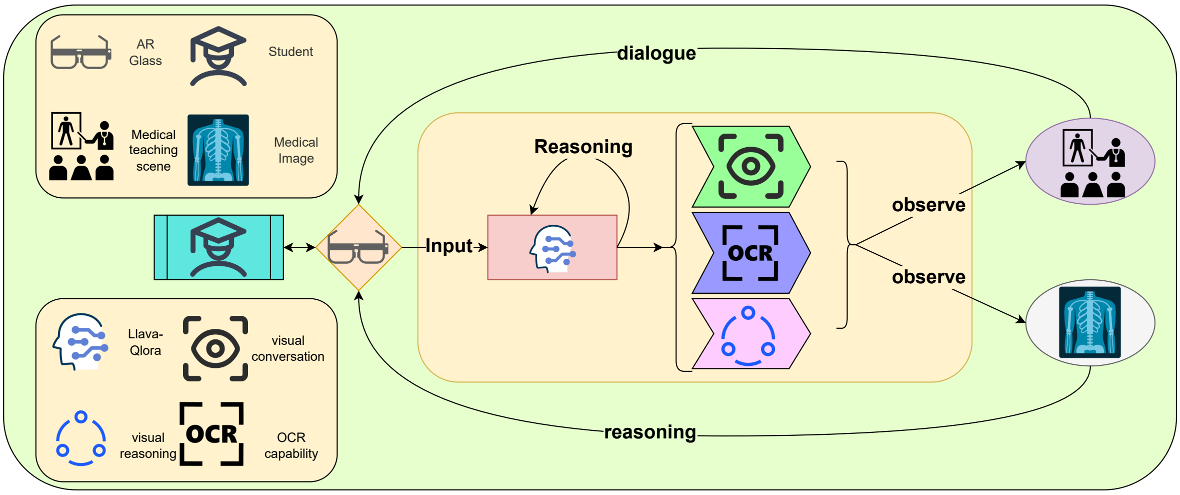

Figure 5: **ARLMT-AR flowchart.** This flowchart illustrates an augmented reality (AR) augmentation framework for medical image analysis. The LLaVA-QLoRA module processes medical images, using CLIP-ViT-L-336px as a visual encoder, while the online VQA component handles real-time diagnostic queries. The reasoning module further refines insights from the VQA to support decision-making. Doctors are equipped with AR devices with front-facing cameras that overlay diagnostic data onto real-world visuals. The ARLMT-AR agent enables AR interaction through intelligent rings to provide enhanced support for doctors to analyze medical images.

Reviewer 3 Comment 5

We note you have included a table to which you do not refer in the text of your manuscript. Please ensure that you refer to Table 2 in your text.

Response

Thank you for your meticulous suggestion. A detailed description of Table 2 has been added in the main text.

Revision

The key advantage of QLoRA in energy-efficient inference stems from its 4-bit integer quantization technique, which significantly reduces memory bandwidth requirements and computation costs. Unlike traditional LoRA fine-tuning, which maintains the original model's precision while introducing additional low-rank adapters, QLoRA directly compresses model parameters into 4-bit representations, minimizing storage and energy consumption. To evaluate QLoRA's performance for edge deployment scenarios, we conducted experiments on an NVIDIA A800 GPU to ensure models run without memory overflow, comparing QLoRA with LoRA and Adapter-based methods. As shown in Table 2, QLoRA demonstrates superior performance, with reduced memory usage (3.2GB for a 7B model), lowe1r energy consumption (1.1W per inference), faster multimodal latency (1.2s), and quicker failure recovery time (98ms) compared to LoRA and Adapter approaches.

Reviewer 3 Comment 6

Please upload a copy of Supporting Information Figure/Table/etc. S1–S3 Figure files which you refer to in your text on pages 19 and 20.

Response

This is helpful suggestion. Due to a misunderstanding of Supporting Information, the legends for Figures 1, 3, 5 and 6 were incorrectly placed in S1, S3, S5 and S6. These have now been adjusted back to their corresponding figure captions in the main text.

Revision

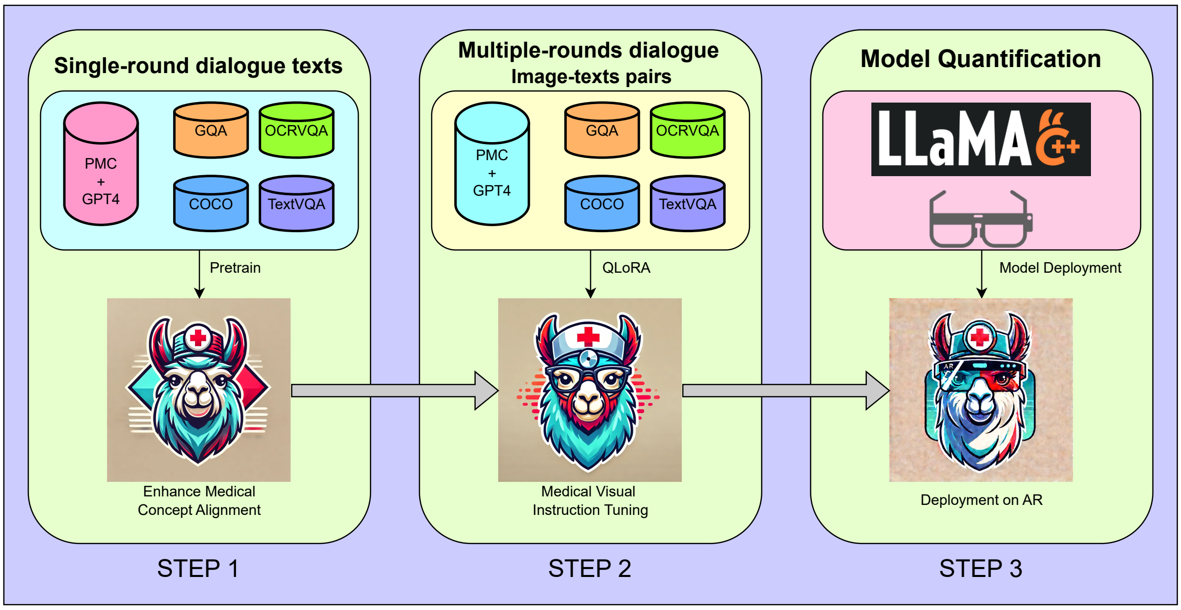

Figure 1: **Full flow chart.** This illustration illustrates a three-step process for developing a medical education-related model for AR deployment. In step 1, a single round of dialogue text from PMC, GQA and other sources is used for pre-training to enhance consistency in medical concepts. The step 2 involves multi-round conversation image-text pairs for medical vision teaching optimization based on QLoRA. Finally, in step 3, the LLamacpp model is quantified and deployed on AR to integrate the previous work for practical medical teaching scenarios.

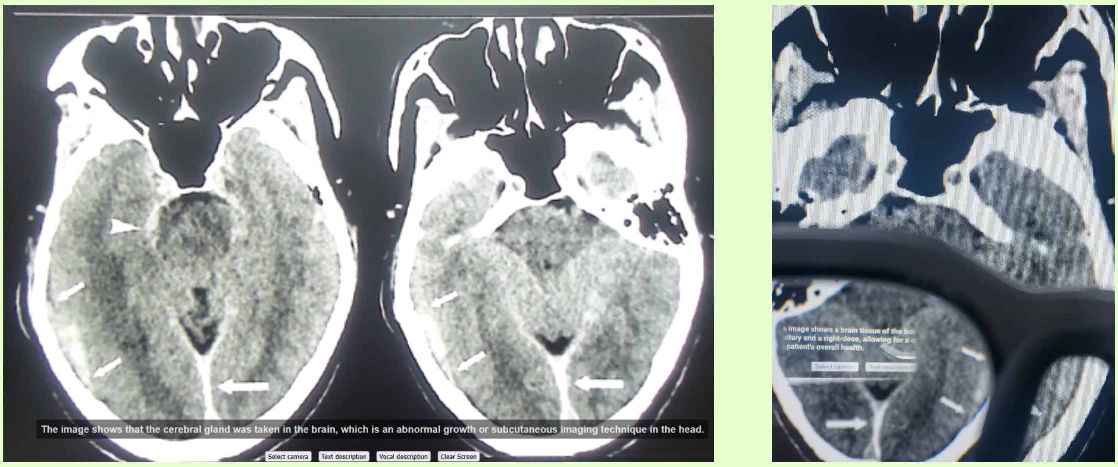

(A) Personal computer simulates human-computer interface

(B) AR glass human-computer interface

Figure 3: **User Interface Layout of the AR Glasses.** User Interface Layout of the AR Glasses. The image consists of two panels, labeled (A) and (B) : (A) The image is the process of implementing human-computer interaction on the simulator of a personal computer; (B) The picture is the process of visual question and answer to the medical image on the display with AR glasses. These panels demonstrate user interface layouts designed for AR glasses that leverage medical imaging data, specifically brain scans in CT or MRI mode, to enhance medical education and diagnostic visualization.

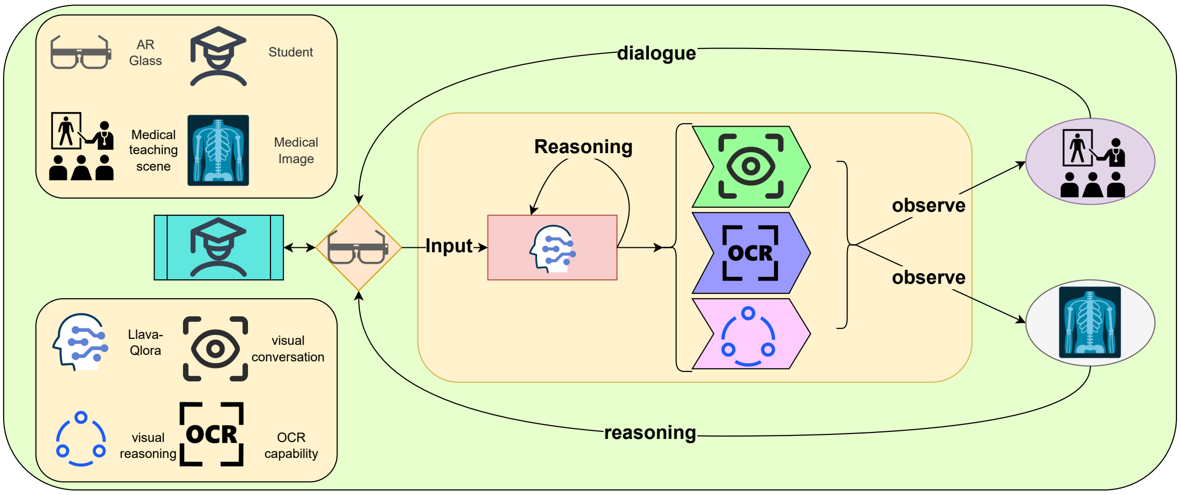

Figure 5: **ARLMT-AR flowchart.** This flowchart illustrates an augmented reality (AR) augmentation framework for medical image analysis. The LLaVA-QLoRA module processes medical images, using CLIP-ViT-L-336px as a visual encoder, while the online VQA component handles real-time diagnostic queries. The reasoning module further refines insights from the VQA to support decision-making. Doctors are equipped with AR devices with front-facing cameras that overlay diagnostic data onto real-world visuals. The ARLMT-AR agent enables AR interaction through intelligent rings to provide enhanced support for doctors to analyze medical images.

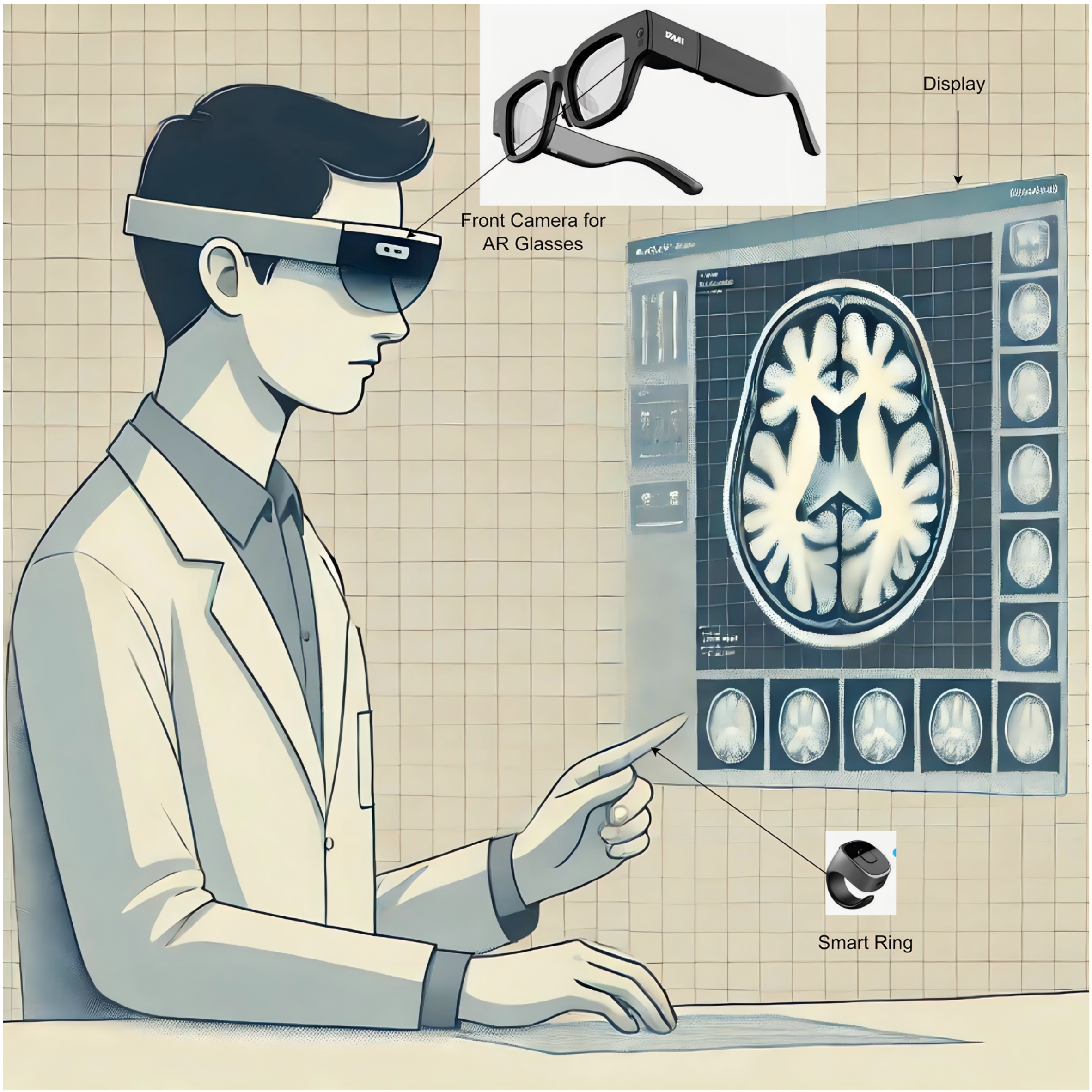

Figure 6: **Online experiment real scene map.** Diagram of the hardware setup for the online experiments, showing the AR glasses (INMO-air2), Philips 247EQ display, front-facing camera and smart ring. The figure visually summarizes the device configuration and the physical arrangement used in the experiment.
