# Supplementary material for: Research on the application of LLaVA model based on QLoRA fine-tuning in medical teaching
Source: PLoS One. 2026 Jul 14;21(7):e0328408. doi: 10.1371/journal.pone.0328408 (PMC13367897; doi:10.1371/journal.pone.0328408)
Supplement: Peer review history

# Peer review: Decision Letter 0

**DOI:** 10.1371/journal.pone.0328408.r001

**Authors:** Ming Liu

7 May 2025

PONE-D-25-16582

ARLMT: application of augmented reality system based on LLaVA in medical teaching and QLoRA fine-tuning

PLOS ONE

Dear Dr. Zhou,

Thank you for submitting your manuscript to PLOS ONE. After careful consideration, we feel that it has merit but does not fully meet PLOS ONE’s publication criteria as it currently stands. Therefore, we invite you to submit a revised version of the manuscript that addresses the points raised during the review process

Please submit your revised manuscript by Jun 21 2025 11:59PM. If you will need more time than this to complete your revisions, please reply to this message or contact the journal office at plosone@plos.org. When you're ready to submit your revision, log on to https://www.editorialmanager.com/pone/ and select the 'Submissions Needing Revision' folder to locate your manuscript file.

Please include the following items when submitting your revised manuscript:

- A rebuttal letter that responds to each point raised by the academic editor and reviewer(s). You should upload this letter as a separate file labeled 'Response to Reviewers'.
- A marked-up copy of your manuscript that highlights changes made to the original version. You should upload this as a separate file labeled 'Revised Manuscript with Track Changes'.
- An unmarked version of your revised paper without tracked changes. You should upload this as a separate file labeled 'Manuscript'.

If you would like to make changes to your financial disclosure, please include your updated statement in your cover letter. Guidelines for resubmitting your figure files are available below the reviewer comments at the end of this letter.

If applicable, we recommend that you deposit your laboratory protocols in protocols.io to enhance the reproducibility of your results. Protocols.io assigns your protocol its own identifier (DOI) so that it can be cited independently in the future. For instructions see: https://journals.plos.org/plosone/s/submission-guidelines#loc-laboratory-protocols. Additionally, PLOS ONE offers an option for publishing peer-reviewed Lab Protocol articles, which describe protocols hosted on protocols.io. Read more information on sharing protocols at https://plos.org/protocols?utm_medium=editorial-email&utm_source=authorletters&utm_campaign=protocols.

We look forward to receiving your revised manuscript.

Kind regards,

Ming Liu

Academic Editor

PLOS ONE

Journal Requirements:

When submitting your revision, we need you to address these additional requirements.

1. Please ensure that your manuscript meets PLOS ONE's style requirements, including those for file naming. The PLOS ONE style templates can be found at

https://journals.plos.org/plosone/s/file?id=wjVg/PLOSOne_formatting_sample_main_body.pdf and

https://journals.plos.org/plosone/s/file?id=ba62/PLOSOne_formatting_sample_title_authors_affiliations.pdf

2. Please note that PLOS ONE has specific guidelines on code sharing for submissions in which author-generated code underpins the findings in the manuscript. In these cases, we expect all author-generated code to be made available without restrictions upon publication of the work. Please review our guidelines at https://journals.plos.org/plosone/s/materials-and-software-sharing#loc-sharing-code and ensure that your code is shared in a way that follows best practice and facilitates reproducibility and reuse.

3. When completing the data availability statement of the submission form, you indicated that you will make your data available on acceptance. We strongly recommend all authors decide on a data sharing plan before acceptance, as the process can be lengthy and hold up publication timelines. Please note that, though access restrictions are acceptable now, your entire data will need to be made freely accessible if your manuscript is accepted for publication. This policy applies to all data except where public deposition would breach compliance with the protocol approved by your research ethics board. If you are unable to adhere to our open data policy, please kindly revise your statement to explain your reasoning and we will seek the editor's input on an exemption. Please be assured that, once you have provided your new statement, the assessment of your exemption will not hold up the peer review process.

4. Please ensure that you refer to Figure 3 and 5 in your text as, if accepted, production will need this reference to link the reader to the figure.

5. We note you have included a table to which you do not refer in the text of your manuscript. Please ensure that you refer to Table 2 in your text; if accepted, production will need this reference to link the reader to the Table.

6. Please upload a copy of Supporting Information Figure/Table/etc. S1 – S3 Figure files which you refer to in your text on page 19 and 20.

**Additional Editor Comments:**

As noted by the two reviewers, there are structural and innovative issues in the paper that require revision.

[Note: HTML markup is below. Please do not edit.]

Reviewers' comments:

Reviewer's Responses to Questions

**Comments to the Author**

1. Is the manuscript technically sound, and do the data support the conclusions?

The manuscript must describe a technically sound piece of scientific research with data that supports the conclusions. Experiments must have been conducted rigorously, with appropriate controls, replication, and sample sizes. The conclusions must be drawn appropriately based on the data presented.

Reviewer #1: Partly

Reviewer #2: Partly

**********

2. Has the statistical analysis been performed appropriately and rigorously?

Reviewer #1: I Don't Know

Reviewer #2: Yes

**********

3. Have the authors made all data underlying the findings in their manuscript fully available?

The PLOS Data policy requires authors to make all data underlying the findings described in their manuscript fully available without restriction, with rare exception (please refer to the Data Availability Statement in the manuscript PDF file). The data should be provided as part of the manuscript or its supporting information, or deposited to a public repository. For example, in addition to summary statistics, the data points behind means, medians and variance measures should be available. If there are restrictions on publicly sharing data—e.g. participant privacy or use of data from a third party—those must be specified.

Reviewer #1: No

Reviewer #2: Yes

**********

4. Is the manuscript presented in an intelligible fashion and written in standard English?

PLOS ONE does not copyedit accepted manuscripts, so the language in submitted articles must be clear, correct, and unambiguous. Any typographical or grammatical errors should be corrected at revision, so please note any specific errors here.

Reviewer #1: Yes

Reviewer #2: Yes

**********

5. Review Comments to the Author

Please use the space provided to explain your answers to the questions above. You may also include additional comments for the author, including concerns about dual publication, research ethics, or publication ethics. (Please upload your review as an attachment if it exceeds 20,000 characters)

Reviewer #1: This subject addressed is within the scope of the journal. However, the manuscript in the present version contains several problems. Appropriate revisions should be undertaken in order to justify recommendation for publication.

• The abstract is written by qualitative sentences. It is need to modify and rewrite based on the most important quantity results from this research. The abstract should be redesigned.

• Please check carefully if all the abbreviations and notations considered in work are explained for the first time when they are used, even if these are considered trivial by the authors. The paper should be accessible to a wide audience.

• The objectives should be more explicitly stated.

• It is better to improve your contributions which are not so clear to show the advantage of your work.

• The novelty of this work must be clearly addressed and discussed in Introduction section.

• The methodology limitation should be mentioned..

• Results. Please interpret the results in a more convenient and clear way, what did you find and why?

• Discussion. Overall, the discussion part is weak. The Discussion should summarize the manuscript's main finding(s) in the context of broader scientific literature and address any study limitations or results that conflict with other published work.

• Conclusion: Some future works should be added to your conclusion. Please elaborate on it a bit more.

Reviewer #2: The organization of the the paper needs to be reconsidered . The current version is not capable of expressing acceptable presentation. Please consider more helpful system such as 2.1, 3.1, 3.1.1 and so on for mentioning the sections and subsections.

**********

6. PLOS authors have the option to publish the peer review history of their article (what does this mean?). If published, this will include your full peer review and any attached files.

If you choose “no”, your identity will remain anonymous but your review may still be made public.

**Do you want your identity to be public for this peer review?**  For information about this choice, including consent withdrawal, please see our Privacy Policy.

Reviewer #1: No

Reviewer #2: No

**********

[NOTE: If reviewer comments were submitted as an attachment file, they will be attached to this email and accessible via the submission site. Please log into your account, locate the manuscript record, and check for the action link "View Attachments". If this link does not appear, there are no attachment files.]

While revising your submission, please upload your figure files to the Preflight Analysis and Conversion Engine (PACE) digital diagnostic tool, https://pacev2.apexcovantage.com/. PACE helps ensure that figures meet PLOS requirements. To use PACE, you must first register as a user. Registration is free. Then, login and navigate to the UPLOAD tab, where you will find detailed instructions on how to use the tool. If you encounter any issues or have any questions when using PACE, please email PLOS at figures@plos.org. Please note that Supporting Information files do not need this step.

# Author comment: Author response to Decision Letter 1

**DOI:** 10.1371/journal.pone.0328408.r002

21 Jun 2025

Reviewer 1 Comment 1:The abstract is written with qualitative sentences. It needs to be modified and rewritten based on the most important quantitative results from this research. The abstract should be redesigned.

Response:We want to thank the reviewer for this comment.We have revised it to include key metrics: a 1.009 second average response time, a 66% memory reduction (from 15.2 GB to 5.1 GB), and 98.3% diagnostic accuracy. These figures highlight the ARLMT system’s performance and benefits.

Reviewer 1 Comment 2:Please check carefully if all the abbreviations and notations considered in the work are explained for the first time when they are used, even if these are considered trivial by the authors.The paper should be accessible to a wide audience.

Response:This is helpful suggestion.We acknowledge the importance of clarity for a broad readership. All abbreviations, such as Augmented Reality (AR), Low-Rank Adaptation (LoRA), Contrastive Language–Image Pre-training Vision Transformer (CLIP-ViT), Quantized Low-Rank Adaptation (QLoRA) and so on, are now defined upon first use.

Reviewer 1 Comment 3:The objectives should be more explicitly stated.

Response:Thanks for the comment. We have revised the introduction to clearly state the objectives: (1) developing an AR-based medical teaching system with AI-driven real-time feedback, and (2) optimizing it for resource-constrained devices using QLoRA.

Reviewer 1 Comment 4:It is better to improve your contributions, which are not so clear, to show the advantage of your work.

Response:Thank you for your valuable feedback. We appreciate your suggestion to improve the clarity of our contributions to better demonstrate the advantages of our work. In response, we have revised the presentation of our key contributions to make them more precise, concise, and explicitly tied to their practical benefits in augmented reality (AR) for medical education. Specifically, we have:(1) Clarified the impact of each contribution by emphasizing how it addresses critical challenges (e.g., resource constraints, integration, and real-time performance).(2) Enhanced readability by refining the language and structure of the contribution list.(3) Added quantitative details and context where applicable to underscore the significance of our innovations. These changes are reflected in the updated manuscript section below. We believe these revisions effectively highlight the novelty and utility of our approach. Please let us know if further adjustments are needed.

Reviewer 1 Comment 5:The novelty of this work must be clearly addressed and discussed in the Introduction section.

Response:Thanks for the comment. We have expanded the introduction to emphasize the novelty: the unique integration of AR and AI, QLoRA optimization for resource-constrained devices, and real-time interactive learning experiences.

Reviewer 1 Comment 6:The methodology limitation should be mentioned.

Response:This is helpful suggestion. We have added a section discussing limitations, including dataset dependency (e.g., PMC-15M), potential latency issues, and hardware constraints of the INMO-Air2 glasses.

Reviewer 1 Comment 7:Please interpret the results in a more convenient and clear way, explaining what was found and why.

Response:We sincerely thank the reviewer for their insightful and valuable feedback, particularly the suggestion to interpret the results in a more convenient and clear manner, with an emphasis on explaining "what was found" and "why." The revised conclusion retains all critical information from the original text, including LLaVA-QLora’s performance metrics (e.g., 79.57% precision on VQA-RAD, 40.78 F1 score on SLAKE), comparisons to baseline models (LLaVA-Med and LLaVA-1.5), and ablation study insights (e.g., 120K model’s superior performance). The content has been rephrased slightly to fit the new structure while maintaining its original intent and technical accuracy.

Reviewer 1 Comment 8:The discussion part is weak. The Discussion should summarize the manuscript’s main finding(s) in the context of broader scientific literature and address any study limitations or results that conflict with other published work.

Response:Thanks for the comment. We have strengthened the discussion by summarizing findings in the context of AR and AI literature, addressing limitations, and comparing results with existing studies.

Reviewer 1 Comment 9:Some future works should be added to your conclusion. Please elaborate on it a bit more.

Response:Thank you for your valuable feedback. The conclusion now includes specific future directions: expanding datasets, improving quantization, minimizing latency, and extending to other medical specialties.

The above responses to the reviewers and editors are only brief text versions. The specific contents are in the "review_response" in the "Attach Files".

Reviewer 2 Comment 1:The organization of the paper needs to be reconsidered. The current version is not capable of expressing acceptable presentation. Please consider more helpful system such as 2.1, 3.1, 3.1.1 and so on for mentioning the sections and subsections.

Response:We want to thank the reviewer for this comment. We greatly appreciate Reviewer #2’s suggestion to improve the manuscript’s organization for enhanced readability and presentation. We agree that a clear hierarchical structure, such as numbered sections (e.g., 2.1, 3.1, 3.1.1), can improve navigation in complex technical papers. However, PLOS ONE’s LaTeX template and style guidelines explicitly restrict the use of numbered section headings, requiring unnumbered \section* and \subsection*commands to maintain a consistent journal format (see PLOS ONE LaTeX guidelines at https://journals.plos.org/plosone/s/latex). To address this constraint while improving clarity, we have taken the following steps: We added a table of contents in the Supporting Information (S4 File) to provide a clear overview of the manuscript’s structure, compensating for the lack of numbered sections.

The above responses to the reviewers and editors are only brief text versions. The specific contents are in the "review_response" in the "Attach Files".

Academic Editor's Comment 1:Please ensure your manuscript adheres to PLOS ONE’s style guidelines, including file naming requirements.

Response:This is helpful suggestion. According to PLOS ONE’s style templates, the title and author affiliations have been formatted correctly. The above responses to the reviewers and editors are only brief text versions. The specific contents are in the "review_response" in the "Attach Files".

Academic Editor's Comment 2:Please note that PLOS ONE has specific guidelines on code sharing for submissions in which author-generated code underpins the findings in the manuscript.

Response:Thanks for this comment. All code have been uploaded to the designated website as per PLOS ONE’s requirements. ARLMT system project code. This file contains the project code for the ARLMT system. The code is available at DOI:https://doi.org/10.6084/m9.figshare.28675511 and can be accessed via https://figshare.com/articles/dataset/_data_zip/28675511 or https://github.com/ZG20250526/ARLMT-Medical-AR-Teaching.

Academic Editor's Comment 3:When completing the data availability statement of the submission form, you indicated that you will make your data available on acceptance.

Response:Thanks for your valuable feedback. All data have been uploaded to the designated website based on the response content.

S1 File. JSON files for LLaVA-Med model training. This data includes JSON files for medical concept alignment and visual capability training of the LLaVA-Med model. The files are available at DOI: https://doi.org/10.6084/m9.figshare.28675511 and can be accessed via https://figshare.com/articles/dataset/_data_zip/28675511. Raw data can be downloaded from https://github.com/microsoft/LLaVA-Med.

S3 File. Fine-tuned and quantized LLaVA-Med model files. This file includes the fine-tuned and quantized model files for the LLaVA-Med model, optimized for efficient deployment on resource-constrained devices. The model files are available at DOI: https://doi.org/10.6084/m9.figshare.28675511 and can be accessed via https://figshare.com/articles/dataset/_data_zip/28675511. Raw model weights can also be downloaded from https://github.com/microsoft/LLaVA-Med.

Academic Editor's Comment 4:Please ensure that you refer to Figure 3 and 5 in your text as, if accepted, production will need this reference to link the reader to the figure.

Response:Thanks for the comment. Due to a misunderstanding of the Supporting Information’s functionality, the explanations and links for Figures 3 and 5 were placed in the Supporting Information section. They have now been adjusted to appear in the main text alongside the figures.

Academic Editor's Comment 5:We note you have included a table to which you do not refer in the text of your manuscript. Please ensure that you refer to Table 2 in your text.

Response:Thank you for your meticulous suggestion. A detailed description of Table 2 has been added in the main text.

Academic Editor's Comment 6:Please upload a copy of Supporting Information Figure/Table/etc. S1–S3 Figure files which you refer to in your text on pages 19 and 20.

Response:This is helpful suggestion. Due to a misunderstanding of Supporting Information, the legends for Figures 1, 3, 5 and 6 were incorrectly placed in S1, S3, S5 and S6. These have now been adjusted back to their corresponding figure captions in the main text.

Attachment Submitted filename: review_response-20250618.pdf

# Peer review: Decision Letter 1

**DOI:** 10.1371/journal.pone.0328408.r003

**Authors:** Ming Liu

1 Jul 2025

Research on the application of LLaVA model based on Q-LoRA fine-tuning in medical teaching

PONE-D-25-16582R1

Dear Dr. Qin,

We’re pleased to inform you that your manuscript has been judged scientifically suitable for publication and will be formally accepted for publication once it meets all outstanding technical requirements.

Within one week, you’ll receive an e-mail detailing the required amendments. When these have been addressed, you’ll receive a formal acceptance letter and your manuscript will be scheduled for publication.

An invoice will be generated when your article is formally accepted. Please note, if your institution has a publishing partnership with PLOS and your article meets the relevant criteria, all or part of your publication costs will be covered. Please make sure your user information is up-to-date by logging into Editorial Manager at Editorial Manager® and clicking the ‘Update My Information' link at the top of the page. If you have any questions relating to publication charges, please contact our Author Billing department directly at authorbilling@plos.org.

If your institution or institutions have a press office, please notify them about your upcoming paper to help maximize its impact. If they’ll be preparing press materials, please inform our press team as soon as possible -- no later than 48 hours after receiving the formal acceptance. Your manuscript will remain under strict press embargo until 2 pm Eastern Time on the date of publication. For more information, please contact onepress@plos.org.

Kind regards,

Ming Liu

Academic Editor

PLOS ONE

Additional Editor Comments (optional):

Based on the feedback returned by the current reviewers, it can be concluded that the paper has addressed the reviewers' main concerns, so the decision is to accept the paper. There are some remaining issues in the paper, and the authors are requested to make corrections in the new submission version.

Reviewers' comments:

Reviewer's Responses to Questions

**Comments to the Author**

1. If the authors have adequately addressed your comments raised in a previous round of review and you feel that this manuscript is now acceptable for publication, you may indicate that here to bypass the “Comments to the Author” section, enter your conflict of interest statement in the “Confidential to Editor” section, and submit your "Accept" recommendation.

Reviewer #3: (No Response)

Reviewer #4: All comments have been addressed

Reviewer #5: (No Response)

**********

2. Is the manuscript technically sound, and do the data support the conclusions?

The manuscript must describe a technically sound piece of scientific research with data that supports the conclusions. Experiments must have been conducted rigorously, with appropriate controls, replication, and sample sizes. The conclusions must be drawn appropriately based on the data presented.

Reviewer #3: (No Response)

Reviewer #4: Yes

Reviewer #5: Partly

**********

3. Has the statistical analysis been performed appropriately and rigorously?

Reviewer #3: (No Response)

Reviewer #4: N/A

Reviewer #5: (No Response)

**********

4. Have the authors made all data underlying the findings in their manuscript fully available?

The PLOS Data policy requires authors to make all data underlying the findings described in their manuscript fully available without restriction, with rare exception (please refer to the Data Availability Statement in the manuscript PDF file). The data should be provided as part of the manuscript or its supporting information, or deposited to a public repository. For example, in addition to summary statistics, the data points behind means, medians and variance measures should be available. If there are restrictions on publicly sharing data—e.g. participant privacy or use of data from a third party—those must be specified.

Reviewer #3: (No Response)

Reviewer #4: Yes

Reviewer #5: No

**********

5. Is the manuscript presented in an intelligible fashion and written in standard English?

PLOS ONE does not copyedit accepted manuscripts, so the language in submitted articles must be clear, correct, and unambiguous. Any typographical or grammatical errors should be corrected at revision, so please note any specific errors here.

Reviewer #3: (No Response)

Reviewer #4: Yes

Reviewer #5: Yes

**********

6. Review Comments to the Author

Please use the space provided to explain your answers to the questions above. You may also include additional comments for the author, including concerns about dual publication, research ethics, or publication ethics. (Please upload your review as an attachment if it exceeds 20,000 characters)

Reviewer #3: (No Response)

Reviewer #4: The manuscript is suitable for publication after the earlier revision have been done to the best of my knowledge

Reviewer #5: Has the system been tested with medical students or physicians? How satisfied were they?

Expand the description of the data used: number of cases, their diversity, etc.

Add statistical support to the results: such as confidence intervals, p-values, etc.

Clearly analyze the limitations and challenges: such as determining the accuracy of the system with rare cases or blurry images.

How can teachers or students actually benefit from the system?

Clearly analyze the limitations and challenges: such as determining the accuracy of the system with rare cases or blurry images.

Cite studies that address the challenges of deploying large models on limited hardware.

It is recommended to include a table summarizing the differences between QLoRA, LoRA, and Adapter in terms of performance, size, power, and accuracy.

Explain the advantages of using INMO Air2 compared to other AR devices.

Ensure all abbreviations and their definitions are clear.

It is recommended to clearly display contributions to the research.

What are the limitations of the method used? It is also preferable to mention future directions that suggest improvements to the method in future work.

**********

7. PLOS authors have the option to publish the peer review history of their article (what does this mean?). If published, this will include your full peer review and any attached files.

If you choose “no”, your identity will remain anonymous but your review may still be made public.

**Do you want your identity to be public for this peer review?**  For information about this choice, including consent withdrawal, please see our Privacy Policy.

Reviewer #3: No

Reviewer #4: **Yes:**  Subday Adeola Ajagbe

Reviewer #5: No

**********
